# Preclinical efficacy and safety assessments of Adult human neural stem cells (AhNSCs) for spinal cord injury

**DOI:** 10.1016/j.toxrep.2025.102048

**Published:** 2025-05-12

**Authors:** Young-Do Kwon, Jeong-Seob Won, Xiangyu Ma, Yoon Jung Choi, Kyoung-Sik Moon, Sang-Jin Park, Eun-Young Gu, Hyeon-Kyu Go, Myung-Jin Kim, Yong-Ho Kim, Geun-Hyoung Ha, Hyun Nam, Chung Kwon Kim, Sungjoon Lee, Sun-Ho Lee, Kyeung Min Joo

**Affiliations:** a Department of Anatomy & Cell Biology, Sungkyunkwan University School of Medicine, Suwon 16419, South Korea; b Medical Innovation Technology Inc. (MEDINNO Inc.), Ace High-End Tower Classic 26, Seoul 08517, South Korea; c Center for Regulatory Toxicology Research, Korea Institute of Toxicology, 141 Gaejeongro, Yuseong-gu, Daejeon, South Korea; d Onheal Co., Ltd., Incheon, South Korea; e HLB Biostep Co., Ltd., Incheon, South Korea; f Stem Cell and Regenerative Medicine Center, Research Institute for Future Medicine, Samsung Medical Center, Seoul 06351, South Korea; g Department of Neurosurgery, Samsung Medical Center, Sungkyunkwan University School of Medicine, Seoul 06351, South Korea; h Department of Health Sciences and Technology, SAIHST, Sungkyunkwan University, Seoul 06351, South Korea; i Regenerative Medicine Center, Seoul National University Bundang Hospital, Seongnam, 13605, South Korea

**Keywords:** Neural Stem Cell, Spinal cord injury, Therapeutic effect, Biodistribution, Toxicity, Tumorigenic potential

## Abstract

Spinal cord injury (SCI) is a severe and devastating condition that leads to irreversible damage to neural tissues, creating significant medical, economic, and social challenges. The ability to differentiate into multiple neural cell types and to regulate immune response makes neural stem cells (NSC) a promising strategy for treating SCI. In this study, we investigated the therapeutic potential, safety profile, and tumorigenic risk of intrathecally transplanted adult human neural stem cells (AhNSCs) produced under clinical-grade standards in a Good Manufacturing Practice (GMP) facility, in rat SCI models, thereby laying the foundation for future clinical trials. Functional tests, including the Basso, Beattie, and Bresnahan (BBB) locomotor rating, rotarod, and von Frey tests, showed significant improvements in motor function and mechanical sensitivity in rats with SCI. Histological analysis revealed reduced tissue loss, glial scar formation, and increased axonal regeneration. Biodistribution studies indicated that the transplanted AhNSCs are primarily localized within the spinal cord, with minimal systemic distribution. Toxicity studies found no significant adverse effects, suggesting a favorable safety profile. Long-term tumorigenicity studies reported no treatment-related deaths or signs of tumor formation in either gender. In conclusion, the study demonstrates that AhNSCs offer promising therapeutic potential for treating SCI, contributing to improved motor function and sensory recovery. These findings support further investigation and potential clinical applications of AhNSCs for treating SCI and related neurological disorders.

## Highlights

• In spinal cord injury, AhNSCs demonstrated significant therapeutic efficacy *in vivo*.
• The transplanted AhNSCs were localized within the spinal cord, with minimal systemic exposure.
• No toxicity-related adverse effects or tumor formation were observed with AhNSCs.
• These results support the safety and potential of AhNSCs for clinical trial development.

## Graphical Abstract

**Figure:**
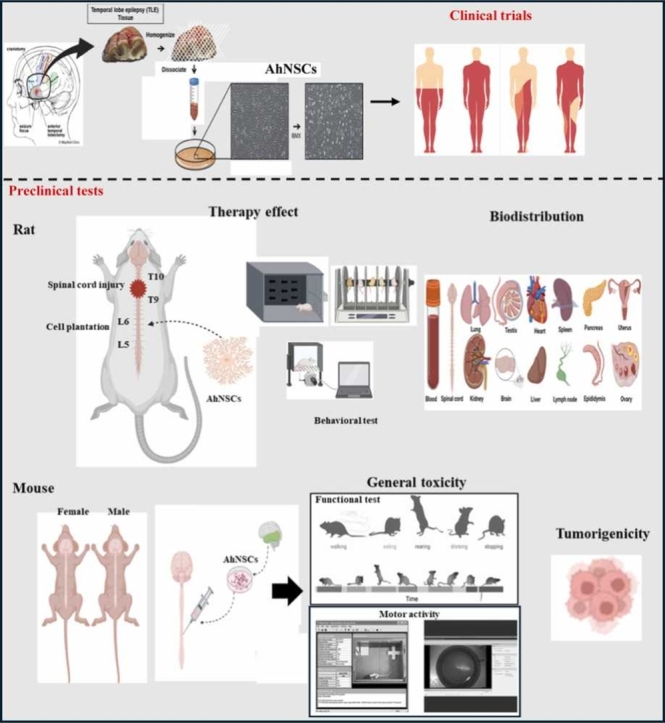

## 1 Introduction

Spinal cord injury (SCI) is a serious devastating injury, resulting in irreversible damage to neural tissues in the spinal cord, and remains a challenging medical, economic, and social problem [2]. Degeneration of spinal cord neurons, as well as axons, and disintegration of neural networks, result in permanent sensory, motor, and autonomic impairments after SCI [11], [3]. The difficulties of SCI therapy are from drastic inflammatory response, lack of neurotrophic factors, inhibitory factors around the spinal cord lesion site, cystic cavities formed at the injured area, and so on [17], [27]. Neural stem cells (NSCs) are multipotent cells capable of self-renewal and differentiation into neurons, oligodendrocytes, and astrocytes, secret neurotrophic factors, and exert anti-inflammatory effects, have been thought to be one of the most attractive strategies for treating SCI [1], [27]. There is massive evidence for the efficacy of NSCs treating SCI in preclinical studies [12], [26]. Moreover, NSCs from different sources such as fetal NSCs and pluripotent stem cells have reached clinical stages [9], [7].

Since no NSC therapeutics for SCI have significant therapeutic effects in clinical settings, new cell therapies based on NSCs need to be further developed and verified. In our previous study, we used adult human neural stem cells (AhNSCs), isolated from the discarded surgical specimens of patients with temporal lobe epilepsy, which have greatly affected treating SCI [16]. Patients with temporal lobe epilepsy have to remove the cerebral cortex within the temporal lobe to avoid seizures, therefore, there are no potential dangers for the donor or ethical concerns [30]. However, NSCs have been thought to exhibit tumorigenicity due to their similarity with cancer cells, and the ability to differentiate into both the nervous system and nonneural cells [31], [5]. Since AhNSCs are derived from the brain with epilepsy, they might make unexpected neural signals *in vivo*. In addition, the allogeneic or xenogeneic transplantation of NSCs may elicit immune responses although they have anti-inflammatory effects [6]. As a result, knowing the biosafety of AhNSCs including toxicity and tumorigenic potential is essential for their clinical applications [18], [21], [28].

In a previous study, we evaluated the genetic stability and tumor formation capacity of AhNSCs after long-term expansion *in vitro* and found that there was gene instability can be induced by long-term *in vitro* expansion, nevertheless, it is not sufficient to exert tumor formation capacity [22]. However, the distribution, general toxicity, and tumorigenic potential of AhNSCs manufactured as a clinically applicable form in good laboratory practice (GLP) conditions remain unclear, although the data is essential for clinical trials. In this study, we manufactured AhNSCs in a clinically applicable form in a good manufacturing practice (GMP) facility and assessed their bioeffect and biosafety including distribution, general toxicity, and tumorigenicity after intrathecal transplantation, which is the clinical administration route, *in vivo* using rodent animal models in GLP conditions. *In vitro* expansion of AhNSCs was performed until *in vitro* passage 5 to avoid genetic instability. This procedure can be used to develop clinical trials to examine the biosafety and bioeffects of stem cells in SCI patients.

## 2 Materials and methods

### 2.1 Cell culture

AhNSCs were prepared at MEDINNO Inc. (Seoul, South Korea), which produces Good Manufacturing Practice (GMP)-grade cell therapy. Briefly, temporal robe surgical samples were obtained upon consent from patients with temporal lobe epilepsy [13]. The master cell banks (MCBs) of AhNSCs were established after *in vitro* passage 4 using the primary culture methods of the previous report [20]. The drug substance (DS) of AhNSCs was expanded *in vitro* for 1 passage from the MCBs. The final drug product (DP) was passage 5. AhNSCs from a single donor were used in this study. For quality control (QC), AhNSCs were tested and confirmed positive for CD29, CD44, CD140b, and Nestin and negative for CD11b, HLA-DR, CD34, CD45, CD19, and CD31. AhNSCs were suitable for mycoplasma, adventitious virus contamination, endotoxin, and cell viability tests. In the tumorigenicity study, human uppsala 87 malignant glioma (U87MG) cells (KIT, Daejeon, South Korea) were used as the Positive Control [14].

### 2.2 Animal husbandry and maintenance

The efficacy and distribution study used Sprague-Dawley (SD) rats from Koatech (Pyeongtaek, South Korea). Athymic nude rats in the general toxicity study were purchased from Envigo RMS Inc. (Indianapolis, IN, USA). The tumorigenicity study used Balb/c nude mice from Orient Co. (Seongnam, South Korea). The rodents were housed in rooms maintained at 23 ± 3 °C, relative humidity of 50 ± 10 % with a 12-h (hr) light-dark cycle, and 10–20 air changes per hr. The animals were housed socially in polysulfone cages with Aspen chip bedding (Hana-biotech, Pyeongtaek, South Korea) and allowed access to sterilized tap water and rodent chow (Lab Diet® #5053; PMI Nutrition International, Bloomington, MN, USA) ad libitum. The experiment was conducted in facilities approved by the Association for Assessment and Accreditation of Laboratory Animal Care (AALAC) International. The animal studies were approved by the Institutional Animal Care and Use Committee (IACUC, approval number: 21-KE-227, 21-KE-444) and performed according to the Animal Experimentation Policy of Knotus Co., Ltd. (Incheon, South Korea).

### 2.3 Spinal cord injury (SCI) modeling

Eight-week-old SD rats were anesthetized for 3 min with a 5 % isoflurane (Ifran™, Hana Pharm, Seoul, South Korea) to oxygen mixture. Anesthesia was maintained at 2.5–3 % isoflurane as needed during surgery. After shaving and incision, laminectomy was performed for the exposure of thoracic spinal cords (T9 plus half T10). Then, SCI was induced by dropping a pendulum weighing 10 g on the exposed dura from a height of 25 mm through a stainless straw. After contusion, the muscle, subcutaneous layer, and skin were sutured according to their anatomical layers. Bladder compression was performed once or twice daily until spontaneous micturition was achieved.

### 2.4 Cell transplantation

Rodents received intrathecal transplantation into L5-L6 interspace according to the previous report [16]. Briefly, under deep isoflurane anesthesia, rodents were placed on the operating table in a prone position. Then, a 50 mL conical tube for rats or a 1.5 mL micro-centrifuge tube for mice were inserted at the front of the knees of the hindlimbs to make the spine bend. The skin and subcutaneous tissue between L5 and L6 were incised. Transplantation using an animal C-arm (Ibis x-ray system, Bergamo, Italy) was performed without muscle removal for rats in the efficacy and distribution test. When AhNSCs were directly transplanted using a 31 G insulin syringe (Hamilton Company, Reno, NV, USA) in the general toxicity and tumorigenicity test, muscles of L5-L6 interspace were carefully removed. After ligamentum flavum was identified, L5 and L6 spinous processes were physically separated, and then AhNSCs were transplanted into the lumbar cistern via the ligamentum flavum. After transplantation, the injection needle was maintained for 1 min to prevent leakage. In the efficacy study, immunosuppression was performed using cyclosporine A (CSA, Chong Kun Dang Pharmaceutical Corp, Seoul, South Korea), which was subcutaneously administered daily from 24 hrs before transplantation to sacrifice. The concentration of CSA was halved every 7 days from 10 mg/kg. When the concentration reached 2 mg/kg, the same concentration of CSA was injected until sacrifice.

### 2.5 Basso-beattie-bresnahan (BBB) test

Rats were placed in an open experimental field and allowed to move freely for 5 mins. During this period, the hindlimb motor abilities of the rats were observed, and each rat's movement was evaluated based on the BBB scale. Hindlimb motor function was evaluated weekly until 6 weeks after transplantation, with tests performed at the same time each day and evaluations conducted by investigators blinded to the group.

### 2.6 Rotarod test

Each animal was placed on a rotarod treadmill (JD-A-07RA5, B.S Technolab Inc., Seoul, South Korea) that rotated at 4 rpm initially and consistently accelerated to 40 rpm for 300 s (secs). The time until the rat fell off the rod onto the floor plate was measured. Each animal underwent one trial per week until 6 weeks after transplantation.

### 2.7 Von frey test

Before the test, rats were placed in a transparent acrylic box with a mesh floor for 30 mins to acclimate to the environment. Then, mechanical stimuli were applied through the mesh using a series of von Frey filaments (ranging from 0.6 to 300 g) to the middle of the right hind paw until the filaments bent within 3–5 s. The Pressure Withdrawal Threshold (PWT) was defined as the minimum gram force that elicited responses such as sharp avoids, shakes, and flinches of the feet. The average PWT value of three consecutive tests was calculated for each animal. Each animal underwent one trial per week until 6 weeks after transplantation.

### 2.8 Histological analysis

Paraffin blocks harboring SCI sites were prepared, sectioned (4 μm thick), and then placed on silane-coated microscope slides (Muto Pure Chemicals Co., Ltd., Tokyo, Japan). The slides were heated on a slide warmer (Lab-line Instruments USA, Dubuque, IA, USA) at 65 °C for 30 mins. After deparaffinization and rehydration, Hematoxylin and Eosin staining (H&E) was performed according to the conventional H&E methods. The tissue area lost in the spinal cord was determined in scanned images of H&E sections and then analyzed using ImageJ software (NIH Image, Bethesda, MD, USA).

### 2.9 Quantitative real-time polymerase chain reaction (RT-PCR)

Rats with SCI that received intrathecal transplantation of AhNSCs were sacrificed according to the schedule. Genomic DNA (gDNA) was extracted from homogenized tissues (40–80 µL) or anticoagulant-treated blood (400 µL) using the GeneAll® Exgene Clinic SV mini kit (GeneAll Biotech. Co., LTD., Seoul, South Korea) or Maxwell 16 LEV Blood DNA kit (Promega, Madison, WI, USA) according to the manufacturer’s instructions. Pooled liver gDNA of SD rats and gDNA extracted from AhNSCs were used as Negative and Positive Control, respectively. Standards and QC samples were prepared using the Negative and Positive Control. PCR was carried out using 100 ng of template in 8 μL of nuclease-free sterile distilled water, 10 μL Power SYBR™ Green Master Mix, and 2 μL of primer mix containing 5 μM of each primer (Alu PCR primers: forward = 5’-GTCAGGAGATCGAGACCAT CCC-3’; reverse = 5’-TCCTGCCTCAGCCTCCCAAG-3’) [21]. Alu RT-PCR was performed on real-time PCR instruments (ViiA™5 or 7 Real-Time PCR System, Applied Biosystems, Foster City, CA, USA) using the following program: 1 cycle at 95 °C for 10 mins, followed by 40 cycles at 95 °C for 15 s and 68 °C for 60 s. The results were analyzed using analysis software (QuantStudio™ Real-Time PCR Software v1.4 and ViiA RUO S/W v 1.2.2 with ViiA SAE module, Applied Biosystems).

### 2.10 Functional observation battery (FOB)

Posture, palpebral closure, tremor, gait/coordinational abnormalities, and convulsion were observed in the cage without any operations. The ease of removal from cage, ease of handling in hand, vocalizations, muscle tone, fur/skin appearance, mucous membrane, palpebral closure, exophthalmos, piloerection, lacrimation, salivation, dehydration, and emaciation were checked during handling when rats were taken out of cages. Righting reflex, palpebral closure, posture, gait/coordinational abnormalities, tremor, convulsion, muscle spasms/fasciculation, respiration ease, rate of respiration, arousal, vocalization, rearing, diarrhea, and polyurea were monitored using an Open Field (85 × 59 cm, height 20 cm) for 2 mins. Approach and touch response, auditory stimulus, tail pinch, and pupillary response were checked. Grip strength was measured with a grip strength meter (model GS3, BIOSEB, Vitrolles, France), and the average of three measurements for both forelimbs and hindlimbs was calculated. To evaluate landing foot splay, ink was applied to the hind paws, and then animals were dropped from a height of approximately 30 cm onto a recording sheet placed on the floor. The average of two measurements between the centers of the paw was determined. Body temperature was checked in the rectum.

### 2.11 Motor activity (MA)

Continuous movement was measured using an Etho Vision XT Version 15 (Noldus Information Technology B.V., Netherlands) for 60 mins, divided into six 10-min intervals. The travel distance (cm), travel velocity (cm/sec), and resting time (sec) were calculated.

### 2.12 Statistical analysis

All data are presented as mean ± standard deviation (SD). Data was analyzed using GraphPad Prism 7.04 (GraphPad Software Inc., San Diego, CA, USA) or Pristima System (Xybion Medical System Co., Princeton, NJ, USA). In studies with three or more groups, Bartlett’s test was used to assume equal variances. Then, homoscedastic data was tested with one-way analysis of variance (ANOVA), and differences between groups were analyzed with Dunnett's Test. Heteroscedastic data were tested by the Kruskal-Wallis T-test, and differences between groups were analyzed by Dunn's Rank Sum Test. P < 0.05 were considered as significant.

## 3 Results

### 3.1 Characterization and identification of AhNSCs

In this study, AhNSCs were successfully isolated and cultured. The marker expression of AhNSCs was analyzed using flow cytometry, revealing positive expression for CD29, CD44, CD140b, and Nestin, while CD11b, HLA-DR, CD34, CD45, CD19, and CD31 were negative (Supplementary Fig. 1A). To evaluate the in vitro differentiation potential of AhNSCs, AhNSCs were cultured in a differentiation condition, and then analyzed by immunocytochemistry. Neural and glial cell markers were used to confirm differentiation (neurons: Tuj1; astrocytes: GFAP; oligodendrocytes: O1)

(Supplementary Fig. 1B). The results indicated that AhNSCs retain key characteristics of neural stem cells (NSCs) and have the potential to differentiate into multiple neural lineages.

### 3.2 AhNSCs demonstrated therapeutic efficacy on SCI

**Fig. 1:**
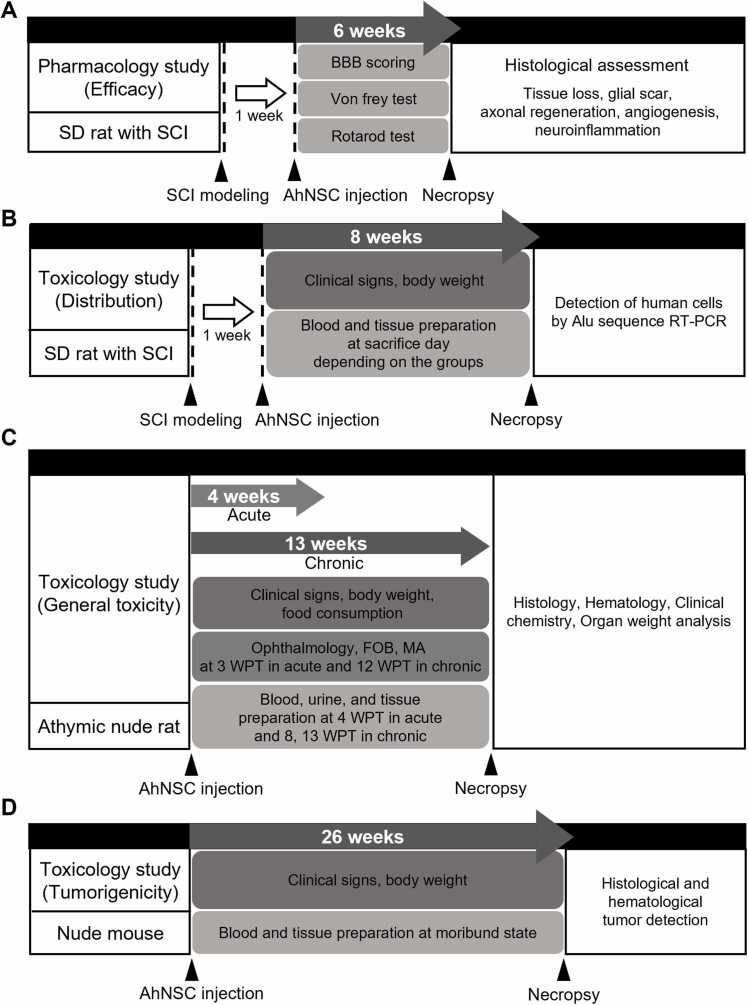
Scheme of *in vivo* preclinical tests. (A) Experiment scheme of preclinical efficacy test. (B) Experiment scheme of preclinical distribution test. (C) Experiment scheme of preclinical general toxicity test. (D) Experiment scheme of preclinical tumorigenicity test. FOB, functional observational battery; MA, motor activity; WPT, weeks post-transplantation.

**Fig. 2:**
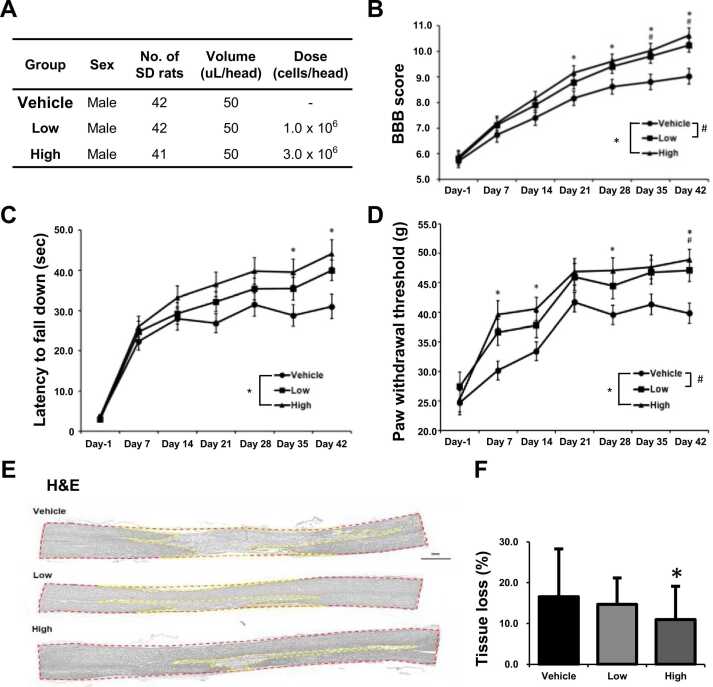
Preclinical therapeutic effects of AhNSCs in SCI. (A) Experimental groups and their conditions were summarized. (B) The BBB scores were evaluated weekly until 6 weeks after intrathecal transplantation of AhNSCs. (C) The motor function was evaluated by the Rotarod test weekly until 6 weeks after intrathecal transplantation of AhNSCs. (D) The sensory function was assessed by the Von Frey test weekly until 6 weeks after intrathecal transplantation of AhNSCs. (E) Tissue loss was measured histologically at 6 weeks after intrathecal transplantation of AhNSCs. The red boxes represent spinal cords, and the yellow lines indicate the areas of tissue loss. (F) The percentage of the tissue loss areas in the spinal cords was calculated and compared. * , P < 0.05, compared with the vehicle group; #, P < 0.05, compared with the vehicle group.

The experiment design is shown in Fig. 1. To examine the therapeutic effects of AhNSCs for SCI, 0 (Vehicle), 1 × 10^6^ (Low), or 3 × 10^6^ (High) AhNSCs in 50 μL DMEM/F12 were transplanted into the intrathecal space between L5 and L6 of SCI rat animal models at 1 week after injury (Figs. 1A and 2A). The BBB score was measured to assess the functional recovery once a week. A significant recovery of motor functions was observed after 3 weeks post-transplantation (WPT) of AhNSCs, especially for the high-dose group (Fig. 2B). In Rotarod tests, the high group also exhibited a significantly longer latency time to fall compared to the vehicle group from 4 WPT (Fig. 2C). In the von Frey test evaluating the neuropathic pain, allodynia, there was also a significant improvement after injecting AhNSCs (Fig. 2D). To confirm the therapeutic effects within the injured spinal cord, tissue loss, glial scar formation, axonal regeneration, neuroinflammation, and angiogenesis were analyzed histologically at 6 (6 WPT). Compared to the vehicle group, the treatment group showed significant decreases in tissue loss (Fig. 2E and F) and glial scar formation (Supplementary Fig. 2A and B), as well as a significant increase in axonal regeneration (Supplementary Fig.2C-E). Additionally, a significant decrease in inflammatory response (Supplementary Fig. 2F-H) was observed, and increased angiogenesis was detected in the AhNSCs-injected group, although it was not statistically significant (Supplementary Fig. 2I-K). These findings suggest that intrathecal transplantation of adult human neural stem cells (AhNSCs) has significant therapeutic effects on spinal cord injury (SCI).

### 3.3 AhNSCs mainly remained in the spinal cord after transplantation

**Fig. 3:**
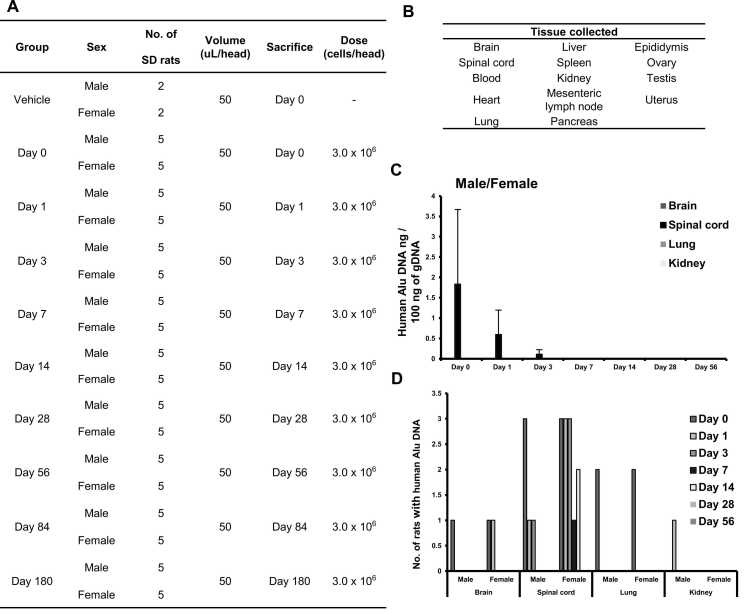
Distribution of AhNSCs in preclinical SCI models. (A, B) Experimental groups, their conditions, and tissues examined were summarized. (C) The amount of human Alu DNA was detected in the 100 ng of gDNA from each organ in each rat by quantitative RT-PCR. (D) Numbers of rats that had human Alu DNA were calculated for each organ.

The distribution of AhNSCs in different tissues was analyzed by quantitative RT-PCR of human-specific Alu-sequence using the SD rats with intrathecal injection of 3 × 10^6^ AhNSCs 1 week after SCI was induced (Fig. 1B). The sacrifice dates, numbers of rats sacrificed, and organs examined are indicated in Fig. 3A and B. The RT-PCR is a highly sensitive and specific method to discriminate between human cells and rodent cells (Supplementary Table 1) [10], which can quantitatively detect 0.005 ∼ 50 ng of human gDNA in 100 ng of SD rat gDNA. In the spinal cord, human gDNA was detected until 3 days post-transplantation (DPT) (Fig. 3C). Human gDNA was also detected in the brain, lung, and kidney in 0–1, 0, and 1 DPT, respectively (Fig. 3D). However, the amounts of gDNA were much lower than that of the spinal cord (Fig. 3C). The results indicated that AhNSCs primarily remained in the spinal cord with limited systemic exposure, suggesting their safety.

### 3.4 AhNSCs showed little toxicity in rodents

**Fig. 4:**
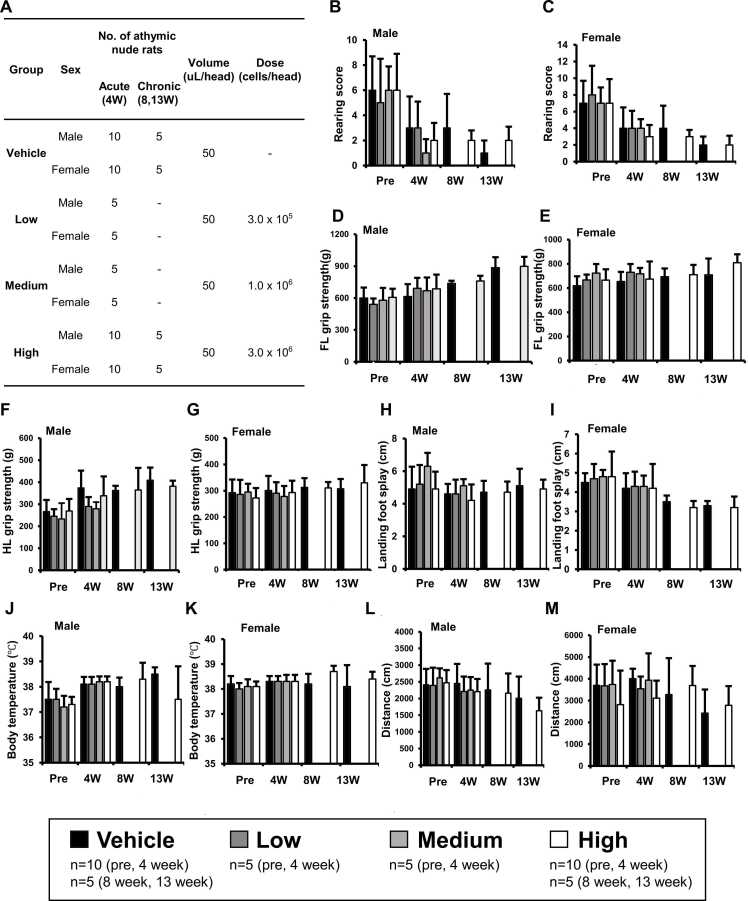
Neurobehavioral effects of AhNSCs in preclinical General toxicity study. (A) Experimental groups, their conditions, and tissues examined were summarized. (B, C) Rearing scores in FOB. (D, E) Forelimb (FL) grip strength in FOB. (F, G) Hindlimb (HL) grip strength in FOB. (H, I) Landing Foot Splay in FOB. (J, K) Rectal body temperature in FOB. (L, M) Movement distance in MA.

Preclinical toxicity studies were performed using athymic nude rats following regulatory authorities including the International Council for Harmonization of Technical Requirements for Pharmaceuticals for Human Use (ICH) (Figs. 1C and 4A). ICH M3 (R2) recommends using immunodeficient animals, as human-derived cells may induce an immune response in the models (2010). Athymic nude rats were randomly allocated to different groups and transplanted once at the dose of 0 (Vehicle), 3 × 10^5^ (Low), 1 × 10^6^ (Medium), or 3 × 10^6^ (High) AhNSCs in 50 μL DMEM/F12 by intrathecal injection (Fig. 4A). General toxicity including mortality, clinical signs, body weights (Supplementary Table 2), food consumption (Supplementary Table 3), ophthalmologic analysis, hematological analysis (Supplementary Table 4), clinical chemistry (Supplementary Table 5), urinalysis, autopsy, pathologic analysis (Table 1), and lymphocyte phenotyping analysis was observed until 4 WTP (acute groups, Fig. 4A). To assess the reversibility, persistence, or delayed occurrence of toxic effects following a 9-week recovery period, additional rats were observed until 13 WTP (for vehicle and high groups only, chronic groups, Fig. 4A). Moreover, to access the potential adverse neurobehavioral effects of AhNSCs in detail, functional observational battery (FOB) and motor activity (MA) tests were applied to 5 animals of both acute and chronic groups, at 4 WTP and 4, 8, and 13 WTP, respectively. FOB included open field observation in cages (posture, palpebral closure, tremor, gait/coordination abnormalities, convulsion) and outside of cages (ease of removal from cage, ease of handling in hand, vocalizations, muscle tone, fur/skin appearance, mucous membrane, palpebral closure, exophthalmos, piloerection, lacrimation, salivation, dehydration, and emaciation), reactivity (approach and touch response, auditory stimulus, tail pinch, and pupillary response), grip strength (both fore and hindlimb), landing foot splay, and body temperature (Fig. 4B-K). MA included movement distance, velocity, and resting time assessments for 60 mins (Fig. 4L and M).

**Table 1:** Microscopic findings in general toxicity study.

		**Acute group (n = 10 for each group)**								**Chronic group (n = 5 for each group)**			
		**Vehicle**		**Low**		**Medium**		**High**		**Vehicle**		**High**	
		**M**	**F**	**M**	**F**	**M**	**F**	**M**	**F**	**M**	**F**	**M**	**F**
**Adrenal gland**	Hemorrhage										1		
**Bone marrow**	Increased cellularity										2		
**Brain**	Cyst	1						1					
	Hydrocephalus	1										1	
**Bladder**	Edema								1				
	Mononuclear cell infiltration		4					1	4				
	Urothelium hyperplasia								1	1			
**Cecum**	Lymphatic vessels dilation												1
	Mononuclear cell infiltration										1		
	Ulceration			1									
**Duodenum**	Mononuclear cell infiltration										1		
**Epididymis**	Mononuclear cell infiltration	9						9		5		5	
	Reduced sperm	9						9				1	
**Esophagus**	Mononuclear cell infiltration								1				
	Squamous cell hyperplasia							1					
**Eyes**	Retinal folds	2							1			1	
	Retinal rosettes	1						1					
**Heart**	Congestion	1						1					
	Mononuclear cell infiltration							1				1	
**Ileum**	Mononuclear cell infiltration										1		
**Jejunum**	Muscular degeneration										1		
	Mononuclear cell infiltration										1		
	Ulceration										1		
**Kidney**	Basophilia in tubules							1					
	Dilation in Pelvis			1		2		2					
	Mononuclear cell infiltration							1			1		1
	Transitional cell hyperplasia							1					
**Liver**	Extramedullary hematopoiesis	1						1	1	1			2
	Fibrosis		2							1			
	Hepatocellular vacuolation								1	1	2		
	Hepatodiaphragmatic nodule						1						1
	Hemorrhage							1					
	Hyperplasia in bile duct		2							1			
	Mononuclear cell infiltration	2					1	1	1	2	1		1
**Lung**	Hemoglobin crystal	1											
	Hemorrhage	2						2					
	Hyperplasia in lymphoid tissue								1	3		4	
	Mononuclear cell infiltration	10						10		5	5	5	5
**Lymphnode**	Dilation									4	2	3	2
	Neutrophil infiltration		1								1		
**Pancreas**	Acinar cell atrophy									1	1		
	Mononuclear cell infiltration											1	

**Pituitary gland**	Cyst in pars distalis										1		
	Rathke's cleft cyst		1										
**Rectum**	Mononuclear cell infiltration										1		
	Inflammatory cell infiltration								1				
**Salivary gland**	Acinar cell atrophy	1								1			
	Acinar cell hypertrophy								1				
	Mononuclear cell infiltration	1											

		**Acute group (n = 10 for each group)**								**Chronic group (n = 5 for each group)**			
		**Vehicle**		**Low**		**Medium**		**High**		**Vehicle**		**High**	
		**M**	**F**	**M**	**F**	**M**	**F**	**M**	**F**	**M**	**F**	**M**	**F**
**Seminal vesicles**	Atrophy	4						3					
	Epithelial hyperplasia									2		2	
**Skin**	Edema			1									
	Epidermal hyperplasia										1		
	Folliculitis	2	1								1		
	Fibrosis			1									
	Hemorrhage			1									
	Inflammatory cell infiltration			1					1	1			1
**Spleen**	Decreased cellularity										1		
	Fibrosis							2					
	Hematopoiesis	2						4		1	2	1	2
	Necrosis		1					1					
**Stomach**	Degeneration	1											1
	Dilation												
	Mononuclear cell infiltration	1	1						2		1	1	1
	Squamous cell hyperplasia	1	2		1			3	3	2	2	1	1
	Squamous cell vacuolation		1						2				
	Ulceration		1				1		1				
**Testes**	Atrophy in tubules	1										1	
	Germ cells degeneration									1			
**Thyroid**	Cystic follicles	1						1	1				
	Follicular cell adenoma								1				
	Ultimobranchial cyst		1					1					
**Uterus/cervix**	Aplasia										1		
	Atrophy										1		

M: Male; F:Female.

A significant decrease in food consumption was observed at 8 DPT in the female medium acute group, 15 DPT in the female high acute group, and 50 DPT in the female chronic group (Supplementary Table 3), which was recovered in the following examination. Hematologic analysis showed a significant increase in the number of RBCs in the female medium acute group and a significant delay in prothrombin time (PT) in the male and female rats of the high acute group (Supplementary Table 4). The male rats of the high acute group and female rats of the high acute group had significant increases in alkaline phosphatase (ALP) and potassium (K), respectively (Supplementary Table 5). The level of creatine kinase (CK) was significantly reduced in the female low acute group (Supplementary Table 5). Significant reduction of the weight of the pituitary gland was found in the female rats of the high acute group (absolute and relative weight to body weight) and the male rats of the high acute group (relative weight to body weight) (Supplementary Table 6 for absolute weight, relative weight not shown). All abnormal findings in the acute groups were not observed in the chronic groups. In the male rats in the chronic group, significant decreases in weight (Supplementary Table 2) and food consumption (Supplementary Table 3) were observed, which were accompanied by significant reductions in the weights of the kidney, liver, and thyroid gland (Supplementary Table 6). Significant increases in the level of chloride (Cl) and total bilirubin (TBIL) were observed in the male and female chronic group, respectively (Supplementary Table 5). Although those abnormalities were statistically significant, they were not accompanied by a pathologic finding. Moreover, they were not dose-dependent and consistent between the acute and chronic groups, which indicates that they might not originate from the toxicities of AhNSCs.

In the autopsy and pathologic analysis, many pathologic changes were found (Table 1). However, they were also observed in the vehicle groups with similar rates and had no dose dependency, which indicates that they might be accidental or spontaneous events. One follicular cell adenoma was found in a male rat of the high acute group. Since no AhNSC-related changes in the thyroid gland were observed in the other rats, it was considered an incidental or spontaneous tumor [4]. No other findings were reported in the preclinical toxicity studies. Altogether, the no-observed-adverse-effect level (NOAEL) of AhNSCs was suggested as 3 × 10^6^ for both male and female rats.

### 3.5 AhNSCs had no tumorigenicity in SCI

**Fig. 5:**
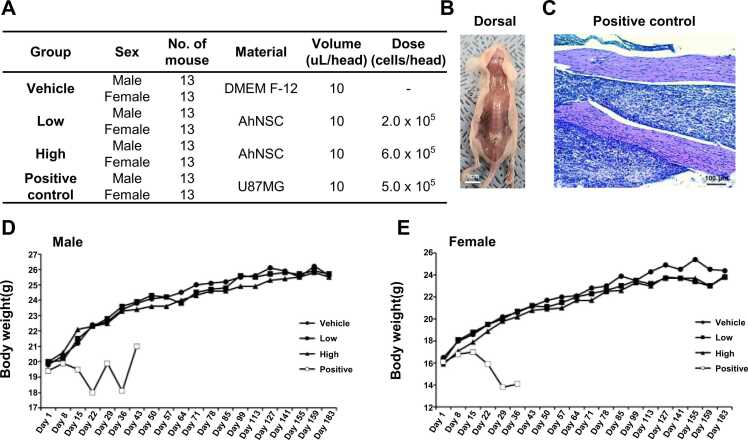
*In vivo* tumorigenicity of AhNSCs. (A) Experimental groups, their conditions, and tissues examined were summarized. (B) After the necropsy, the injection site and the extracted vertebral column were observed. (C) Progressive tumors were confirmed in all animals in the positive control group. (D, E) Body weight was measured to screen the development of tumor.

Tumorigenicity of AhNSCs was assessed by employing immunodeficient animals for a designated observation period to sufficiently monitor the occurrence of tumor formation (6 months) (Fig. 1D) [25]. The animals underwent a single transplantation procedure via intrathecal injection, receiving either 0 (vehicle), 2 × 10^5^ (low), or 6 × 10^5^ (high) AhNSCs in 10 μL DMEM/F12, and 5 × 10^5^ U87MG cells as positive control were transplanted for experimental validity (Fig. 5A) [23]. Tumor formation was evaluated using both macroscopic and microscopic examination methods. Necropsy examinations were performed on dead, accidentally dead, moribund sacrificed, and scheduled sacrificed animals during the observation period (Fig. 1). Following necropsy, the injection site and extracted vertebral column were macroscopically inspected. In cases where gross tumors were not visually apparent, the spinal cord was collected, stained with H&E, and examined under a microscope. In the positive control group, progressive tumors were confirmed in all animals (Fig. 5B). The spinal cords exhibited infiltrative tumor cells, characterized by atypical morphologic features (Fig. 5C). In contrast, no AhNSC-related deaths, clinical symptoms, changes in body weight, or histopathological alterations were observed in either the vehicle or AhNSC transplantation groups (Fig. 5D and E). No evidence of tumorigenesis suggested the safety of AhNSCs.

## 4 Discussion

This study investigated the therapeutic effects and safety including the biodistribution and tumorigenicity of AhNSCs intrathecally transplanted into rat SCI models. Since the intrathecal injection is the administration route to be used in clinical trials of AhNSCs and AhNSCs were manufactured according to the protocols applied to clinical trials, the experimental results in this study could be used as preclinical background data for clinical trials. Additionally, the GLP conditions in this study further enhance the reliability of its results. In this study, we found that AhNSCs significantly promote tissue repair and functional recovery of SCI animal models while they have little toxicity and tumorigenicity, which is adequate to proceed with clinical trials.

AhNSCs have improved the motor and sensory functions of limbs of SCI animal models. The functional recovery was accompanied by the reductions in tissue loss and glial scar formation as well as the increase in axonal and dendrite regeneration in the tissue areas of SCI. These results are consistent with our previous studies demonstrating the therapeutic potential of AhNSCs in treating SCI [16], [19]. Furthermore, AhNSCs may mediate SCI recovery through multiple mechanisms beyond direct neuronal replacement [19]. They secrete neurotrophic factors, modulate immune responses, and create a more permissive microenvironment for endogenous repair. Conventional SCI treatments, such as rehabilitation and pharmacological therapies, primarily focus on symptom management and maintaining the patient's condition, often accompanied by potential side effects. In contrast, AhNSCs offer the potential to treat the SCI itself, facilitating fundamental recovery and presenting a clear advantage over existing therapies. However, challenges remain in translating these findings into clinical applications, including optimizing cell survival, determining the ideal dosage, and understanding long-term effects. Future studies should focus on elucidating these mechanisms and refining therapeutic strategies to achieve successful clinical translation.

Although AhNSCs have the potential to differentiate into neurons and directly replace the lost neurons, the distribution study indicated that most AhNSCs disappeared in the spinal cord within one week after transplantation. If AhNSCs remain at the injury site and differentiate into functional neurons, they might provide neuronal substrates for electrical signals to bridge or circumvent the lesion area. However, experimental results indicated that AhNSCs exert their therapeutic effects via other mechanisms rather than the replacement of damaged tissue. The other mechanisms would be the induction of endogenous tissue recovery and modulation of microenvironments of the damaged spinal cord, which might be mediated by paracrine factors secreted from AhNSCs just after transplantation. In the previous study, we reported the paracrine factors of AhNSCs that might make the therapeutic effects of AhNSCs in SCI [16], [15].

Our previous study [29] using an antibody for human-specific cytoplasm antigen demonstrated that AhNSCs transplanted into the lumbar cistern of SCI animal models were primarily retained within the spinal cord until 5 weeks after injection, with limited systemic exposure to other organs. Accordingly, the distribution study of this study demonstrated that intrathecally transplanted AhNSCs were primarily retained within the spinal cord with limited systemic exposure in another organ. Human-specific Alu sequence was detected in the brain, lung, and kidney. Since the brain and spinal cord are connected organs in the same intrathecal space, AhNSCs transplanted into the lumbar cistern might migrate to the brain via the cerebrospinal fluid (CSF) system. The amount of human-specific Alu sequence detected in the lung and kidney was much lesser than that of the spinal cord and it was detected within 1 day after transplantation of AhNSCs. Regarding AhNSCs transplanted in the spinal cord could not live long, the Alu-sequence might be the gDNA released from dead AhNSCs and then delivered to the organs by circulating blood. Notably, the viability of AhNSCs was higher in female rats than male rats, indicating potential sex-related differences in stem cell survival and retention. The differences might originate from the difference in the immune reactions between female and male rats and the origin of AhNSCs, that is a male donor. These findings align with recommendations from the International Society for Stem Cell Research (ISSCR) regarding the importance of assessing cell migration and retention in cell therapy products [20].

The general toxicity study assessed the safety profile of AhNSCs using immunodeficient athymic nude rats. The study showed no significant adverse effects related to AhNSCs suggesting a favorable safety profile at the tested doses. Most importantly, the potential adverse neurobehavioral effects of AhNSCs were analyzed in detail using FOB and MA tests since AhNSCs were derived from donors who have epileptic seizures. The thorough examinations demonstrated the safety of AhNSCs with no neurobehavioral effects of AhNSCs. However, there were numerous alterations in the general toxicity test that were supposed to be endogenous changes that happened accidentally in this study. This conclusion was based on the lack of dose-dependency, incidence of the alterations in the rats that had only vehicle (Negative control), and spontaneous resolves in the chronic groups. In the general toxicity tests, thymic nude rats were utilized. However, the rats have a partially innate immune system that could react to the xenogeneic AhNSCs. Therefore, the safety of AhNSCs might need to be confirmed using animal models that lack the immune system or have the human immune system [8].

The long-term tumorigenicity study evaluated the potential of AhNSCs to form tumors following intrathecal transplantation. No treatment-related deaths, clinical symptoms, or signs of tumor formation were observed in either gender, which indicates the safety of AhNSCs. Although the safety of AhNSCs was validated using various preclinical examinations in this study, there are still some challenges in translating preclinical studies into the clinical set, including anatomical differences between experimental animal models and human SCI patients and different numbers of transplanted cells in preclinical and clinical studies [24]. For example, in the preclinical tumorigenicity test of this study, tumor formation was monitored for 6 months. However, the expected lifespan of human SCI patients is much longer than that. Therefore, the safety and preliminary efficacy of AhNSCs for SCI should be tested in the phase I clinical trial. In addition, various preclinical examination technologies need to be developed to close the gap between preclinical and clinical studies.

In summary, this study demonstrates the therapeutic potential of AhNSCs for treating SCI, as evidenced by significant improvements in motor function and sensory recovery. This study also provides a comprehensive safety assessment, showing minimal systemic exposure and no significant tumorigenicity, supporting the viability of AhNSC-based therapies for SCI and related neurological disorders. These findings lay the groundwork for further clinical investigation and potential therapeutic applications of AhNSCs.

## Funding

This research was supported by a grant of the Korea Health Technology R&D Project through the Korea Health Industry Development Institute (KHIDI), funded by the Ministry of Health & Welfare, Republic of Korea (grant number: RS-2022-KH129441).

## Author contributions

Kyung Min Joo conceived the project and designed the experiments. Geun-Hyoung Ha, Nam Hyun, Eun-Young Gu, and Yoon Jung Choi analyzed data and managed the project. Young-Do Kwon, Jeong-Seob Won, and Xiangyu Ma conducted the research, established animal models, performed immunohistochemistry, and contributed to manuscript writing. Hyeon-Kyu Go, Myung-Jin Kim, and Yong-Ho Kim carried out toxicity analysis and data analysis. Kyoung-Sik Moon, Sang-Jin Park, Chung Kwon Kim, and Sun-Ho Lee contributed to the isolation and analysis of AhNSCs.

## CRediT authorship contribution statement

**Go Hyeon-Kyu:** Software, Resources, Formal analysis, Data curation. **Gu Eun-Young:** Resources, Formal analysis. **Park Sang-Jin:** Software, Methodology, Formal analysis, Data curation. **Joo Kyeung Min:** Writing – review & editing, Writing – original draft, Visualization, Supervision, Methodology. **Moon Kyoung-Sik:** Software, Project administration, Formal analysis, Data curation. **Lee Sun-Ho:** Visualization, Validation, Supervision, Data curation. **Choi Yoon Jung:** Resources. **Lee Sungjoon:** Resources, Formal analysis, Data curation, Conceptualization. **Ma Xiangyu:** Writing – review & editing, Writing – original draft. **Kim Chung Kwon:** Resources, Project administration, Conceptualization. **Won Jeong-Seob:** Software, Data curation, Conceptualization. **Nam Hyun:** Supervision, Software, Data curation, Conceptualization. **Kwon Young-Do:** Writing – review & editing, Writing – original draft, Visualization, Software, Formal analysis. **Ha Geun-Hyoung:** Supervision, Software, Formal analysis, Data curation, Conceptualization. **joo kyeung min:** Writing – review & editing, Writing – original draft, Visualization, Validation, Supervision, Software, Resources, Project administration, Methodology, Investigation, Funding acquisition, Formal analysis, Data curation, Conceptualization. **Kim Yong-Ho:** Software, Formal analysis, Data curation. **Kim Myung-Jin:** Software, Formal analysis, Data curation.

## Declaration of Competing Interest

The authors declare that they have no known competing financial interests or personal relationships that could have appeared to influence the work reported in this paper.

## Data Availability

Data will be made available on request.

## Footnotes

Appendix A Supplementary data associated with this article can be found in the online version at doi:10.1016/j.toxrep.2025.102048.
