# Supplementary material for: Preclinical efficacy and safety assessments of Adult human neural stem cells (AhNSCs) for spinal cord injury
Source: Toxicol Rep. 2025 May 12;14:102048. doi: 10.1016/j.toxrep.2025.102048 (PMC12149656; doi:10.1016/j.toxrep.2025.102048)
Supplement: Supplementary file 1 — Supplementary material [file mmc1.docx]

**A**


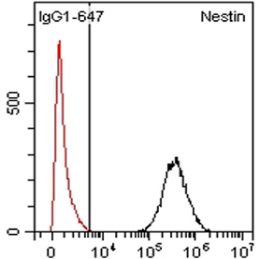

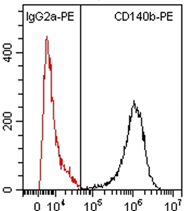

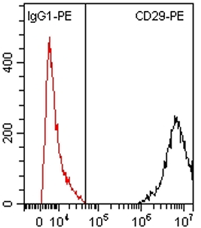

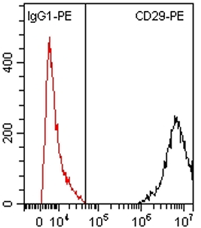


**Isotype IgG**

**99.94%**

**99.06%**

**99.54%**

**99.92%**


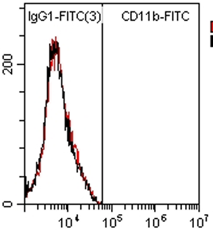

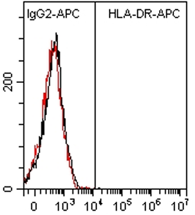

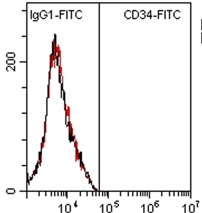

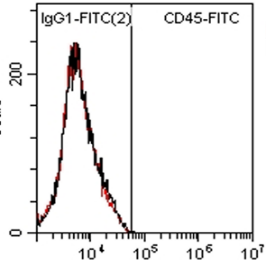

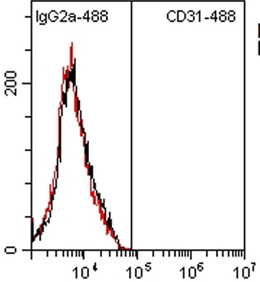

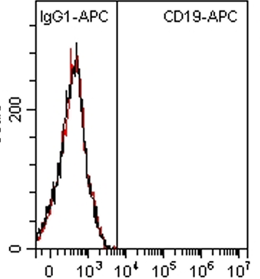


**Indicated cell surface proteins**

**CD31**

**CD19**

**CD45**

**CD34**

**HLA-DR**

**CD11b**

**Nestin**

**CD140b**

**CD44**

**CD29**

c

c

c

c

**0.16%**

**0.18%**

**0.18%**

**0.1%**

**0.35%**

**0.1%**

**B**

**O1**

**Nestin**

**Tuj1**

**GFAP**

**BF**

**
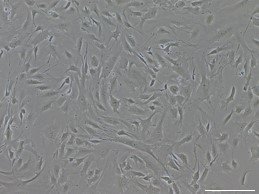
**
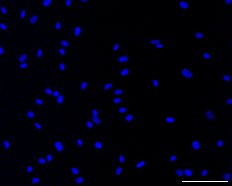

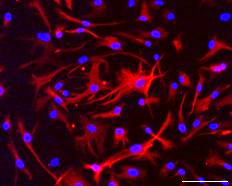

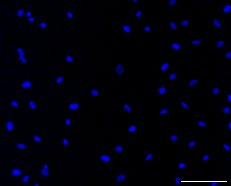

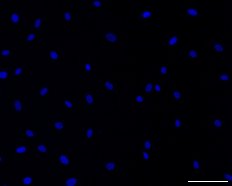


**Undiffer**

**
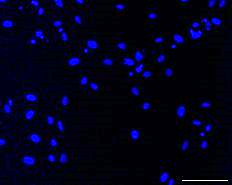

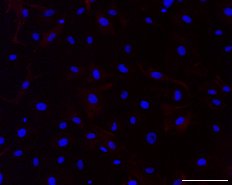

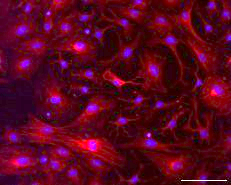

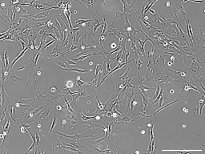

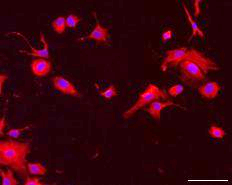
**

**Differ**

**Supplementary Figure 1. Expression of various markers and *in vitro* differentiation potential of ahNSCs.**
(**A**) The expression levels of various markers were analyzed by flow cytometry. AhNSCs were positive for CD29, CD44, and Nestin and negative for CD11b, HLA-DR, CD34, CD45, CD19, and CD31. The number in each panel indicates the percentage of marker-positive cells.
(**B**) The expression of the NSC marker (Nestin) and neural cell markers (neurons: Tuj1; astrocytes: GFAP; oligodendrocytes: O1) was analyzed by immunocytochemistry in undifferentiated (Undiff) and differentiated (Diff) cells. BF: Bright field. Scale bar: 20 µm.

**Tuj1**


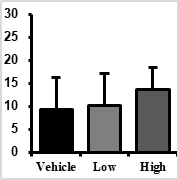


**Tuj1 positive area (%)**

*


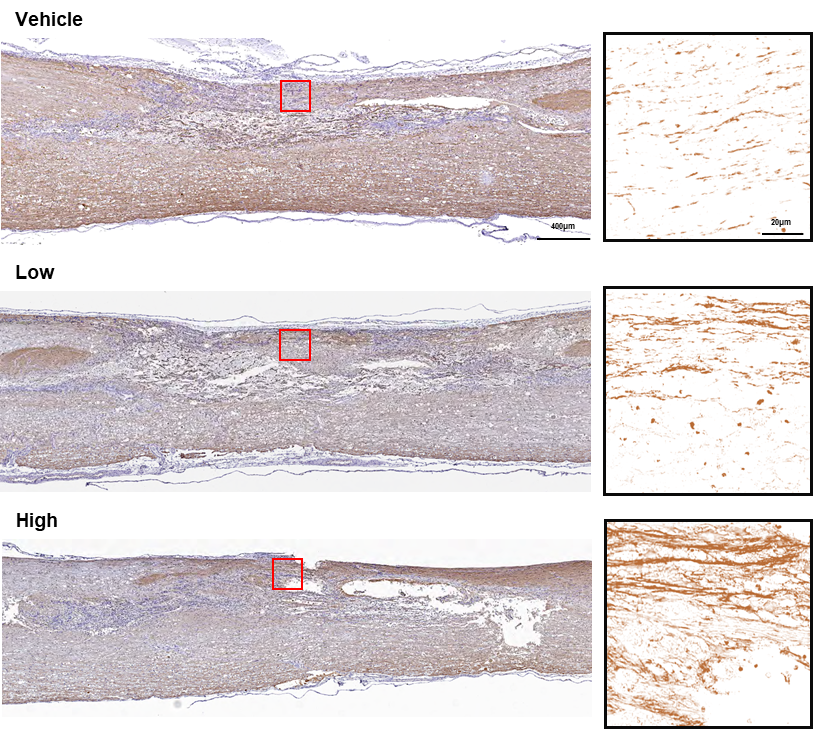

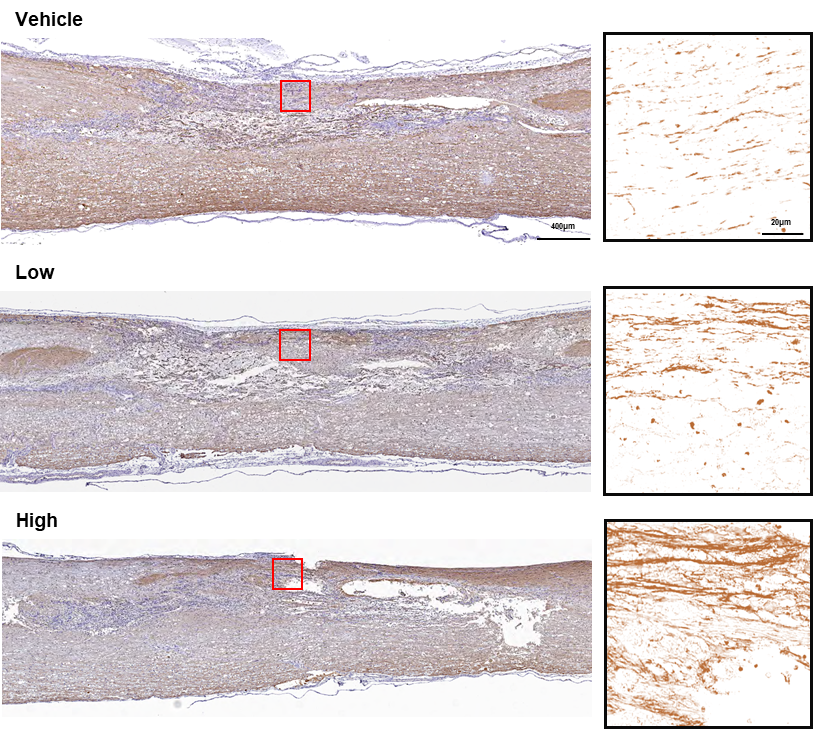

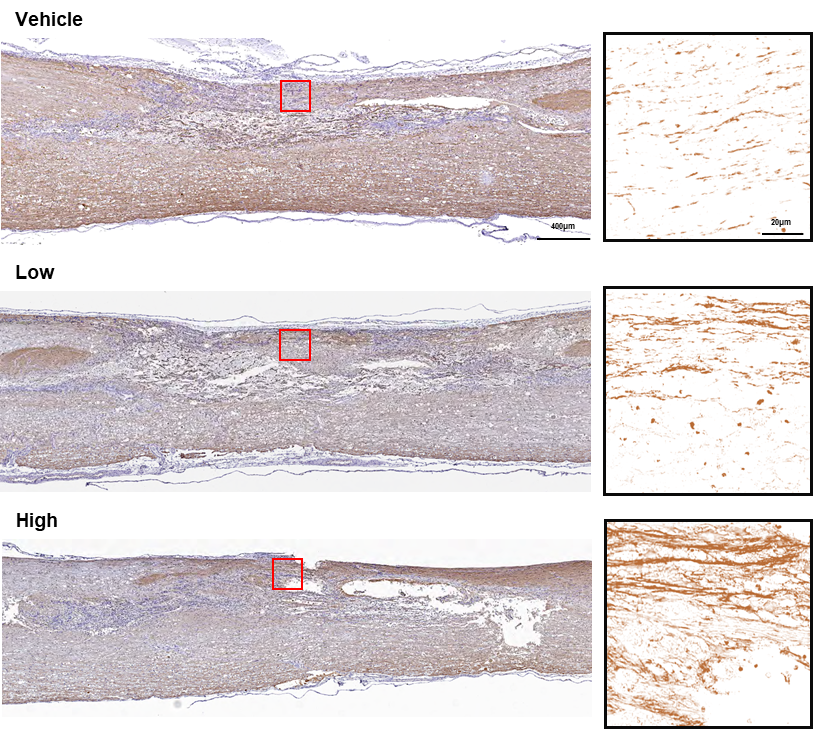

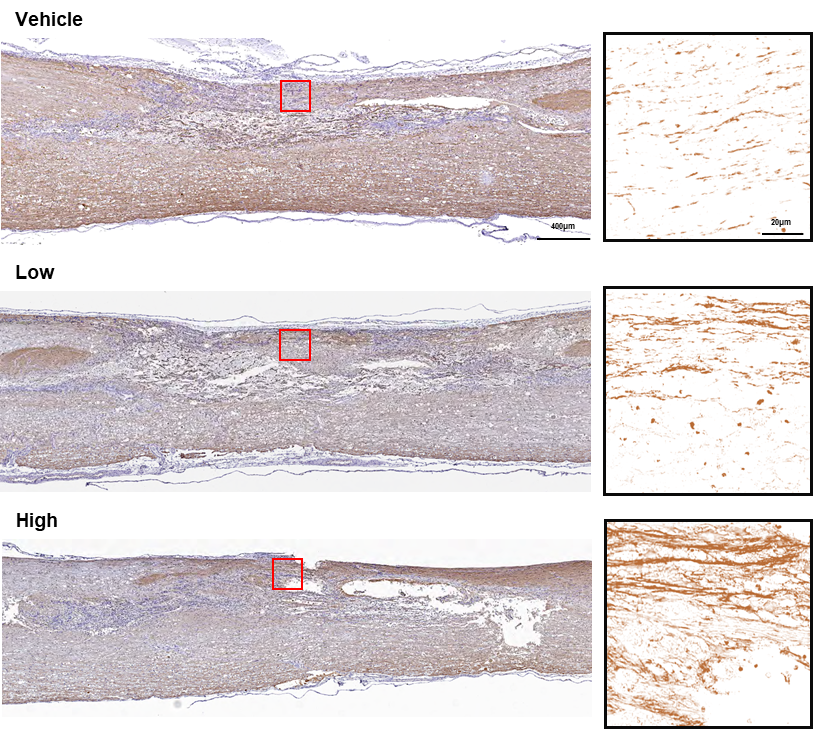


**Vehicle**

**Low**

**High**

**C**

**D**

**E**


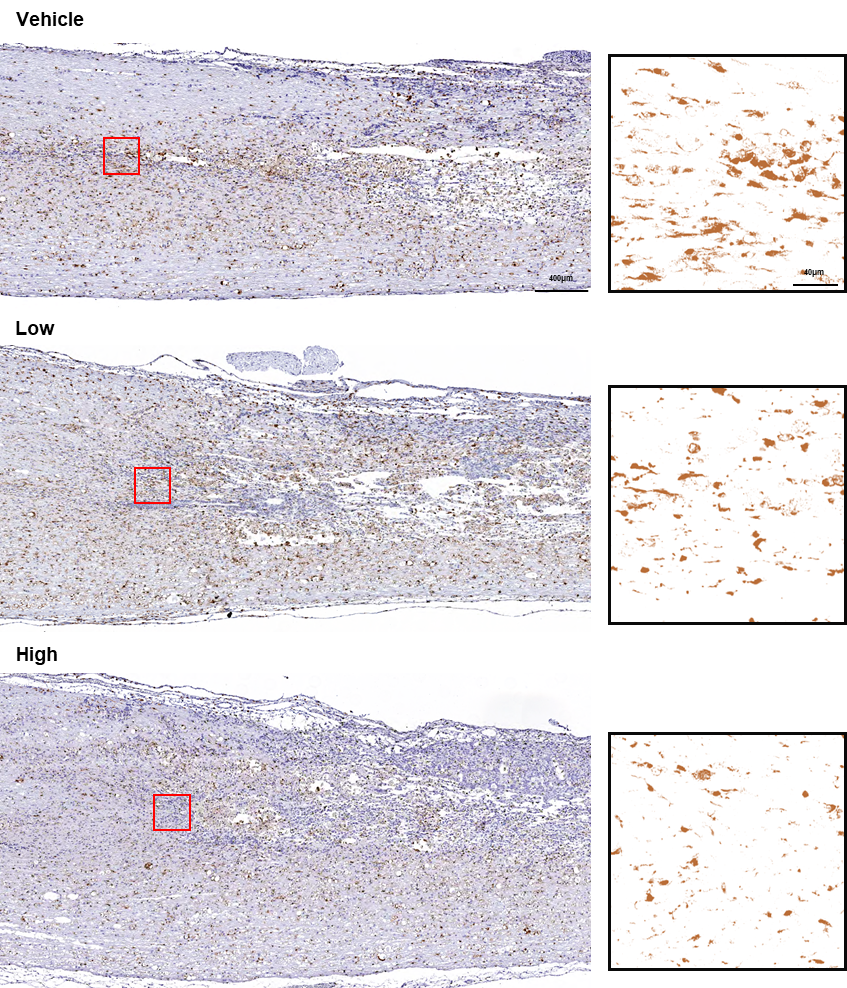

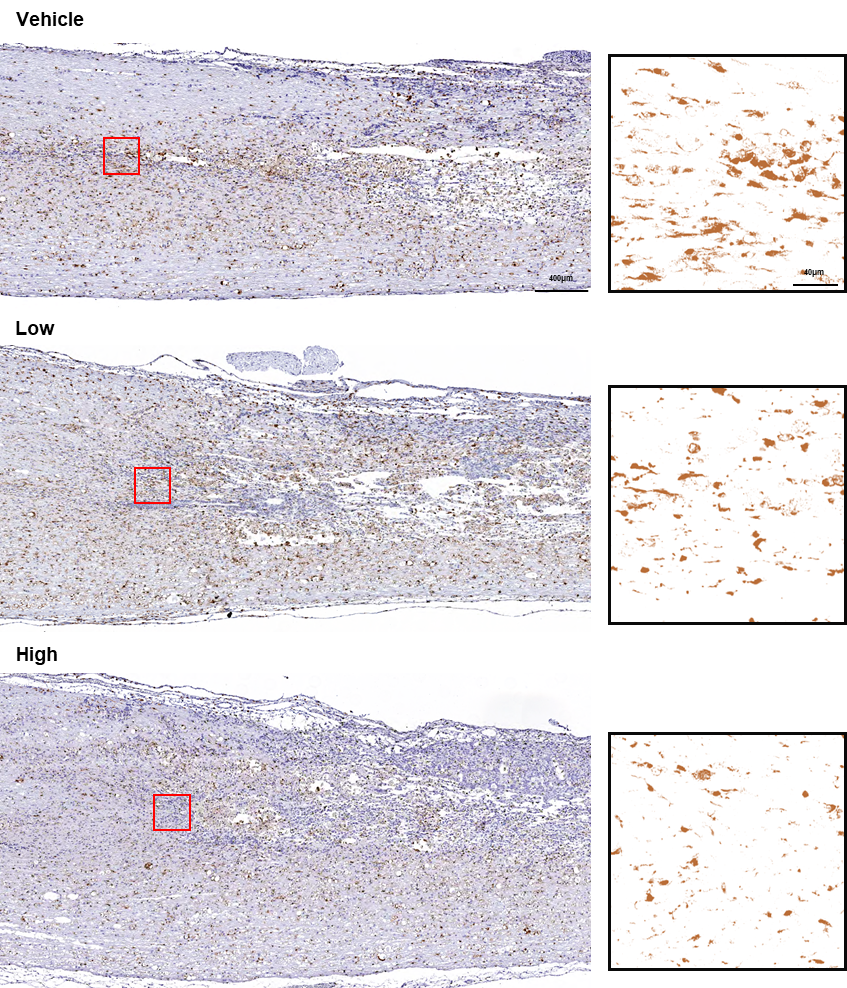

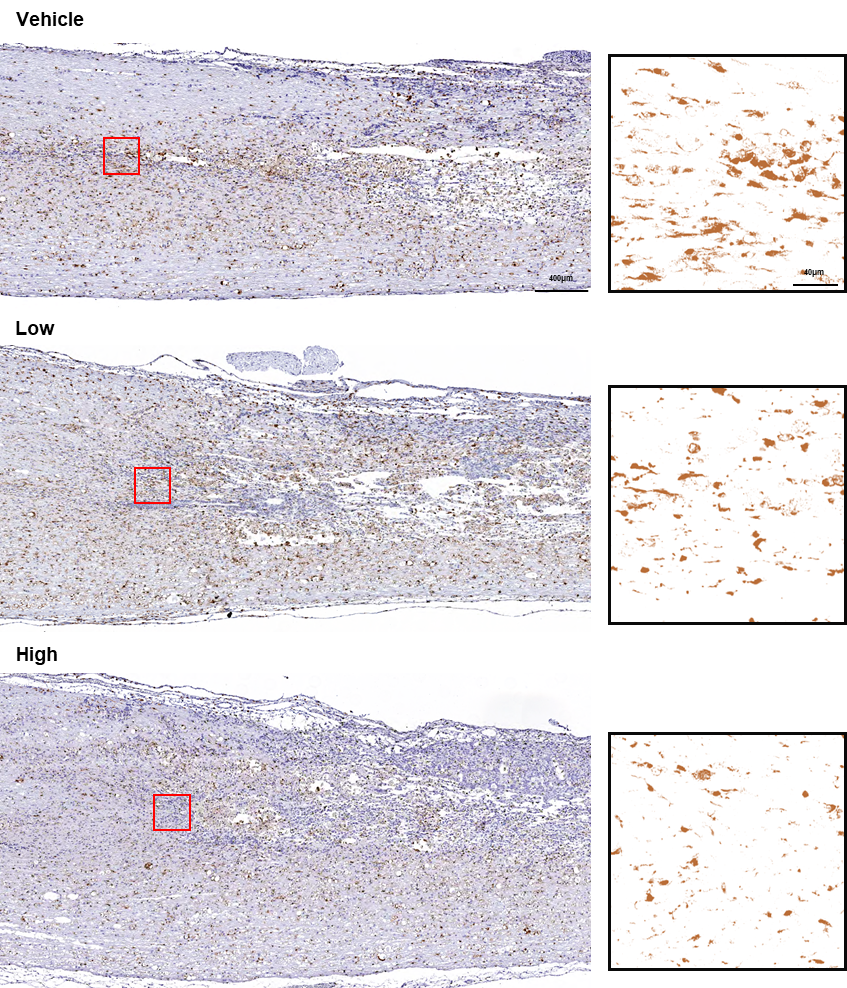


**Vehicle**

**Low**

**High**


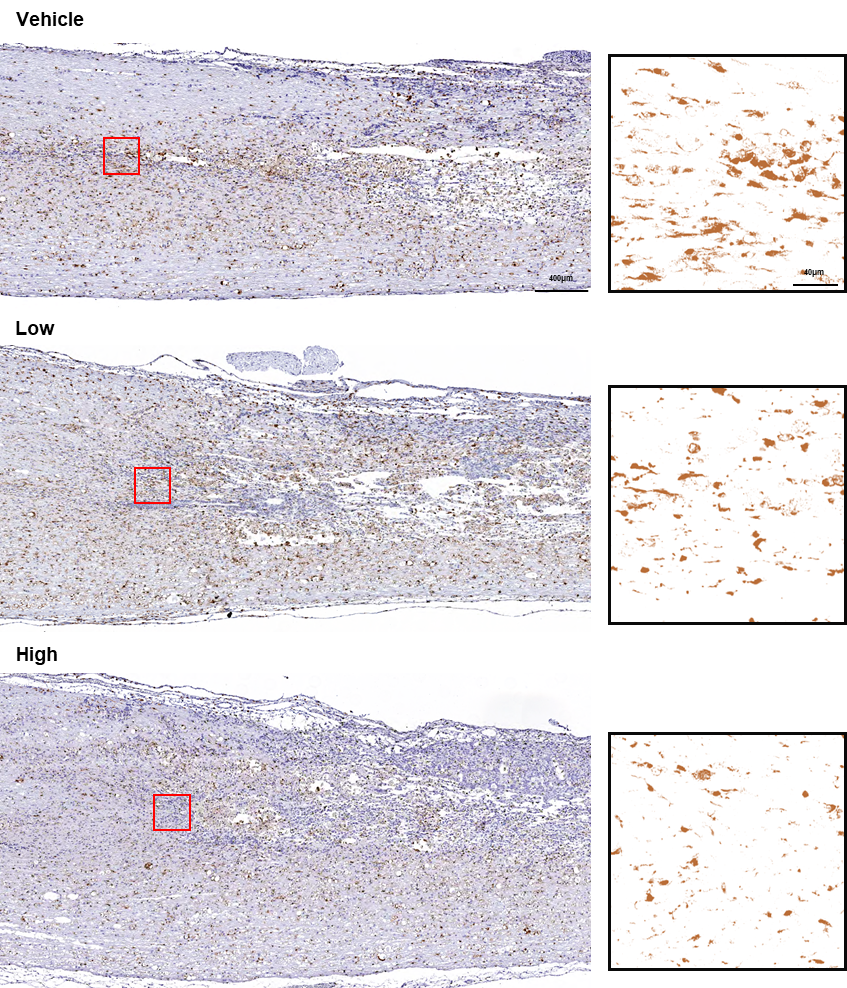


**F**

**G**

**H**


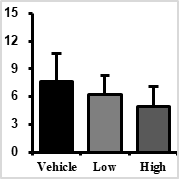


**Iba1 positive area (%)**

***

*


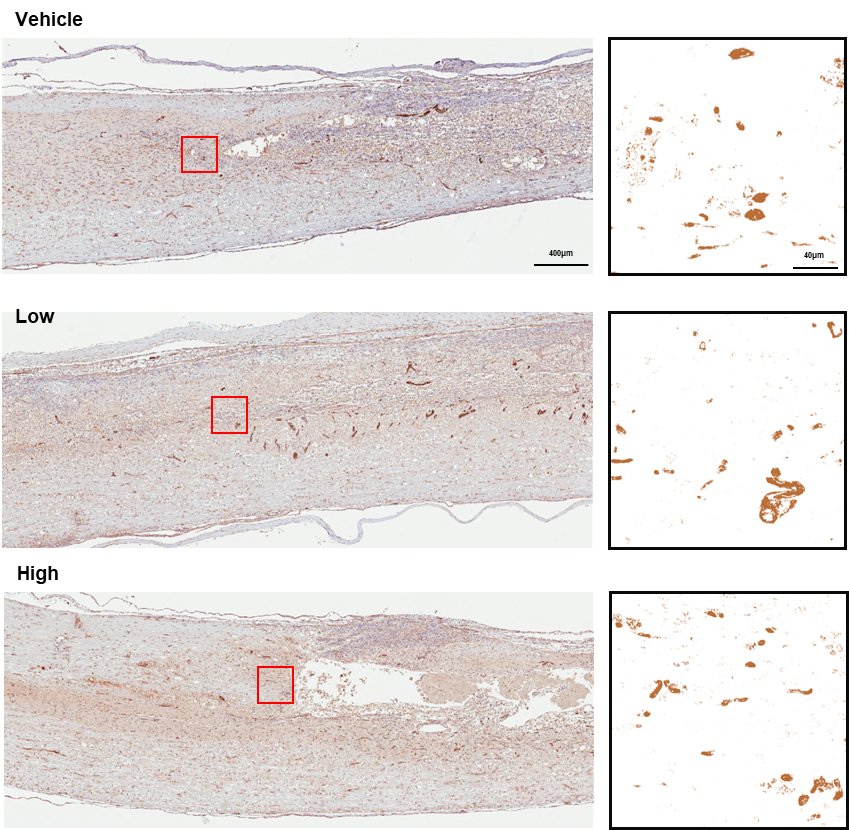

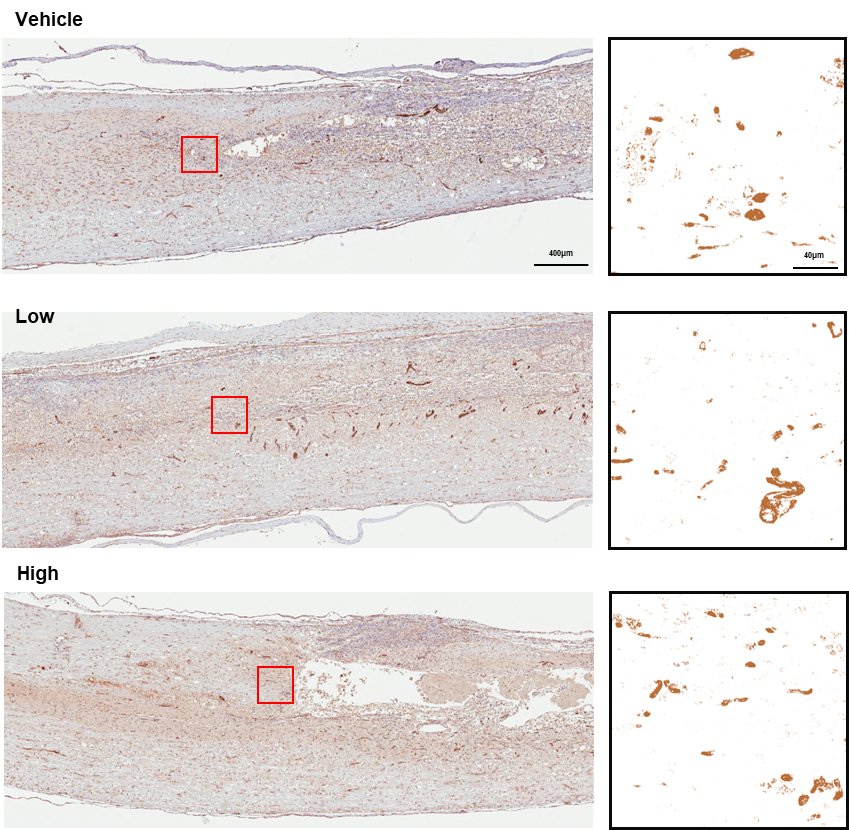

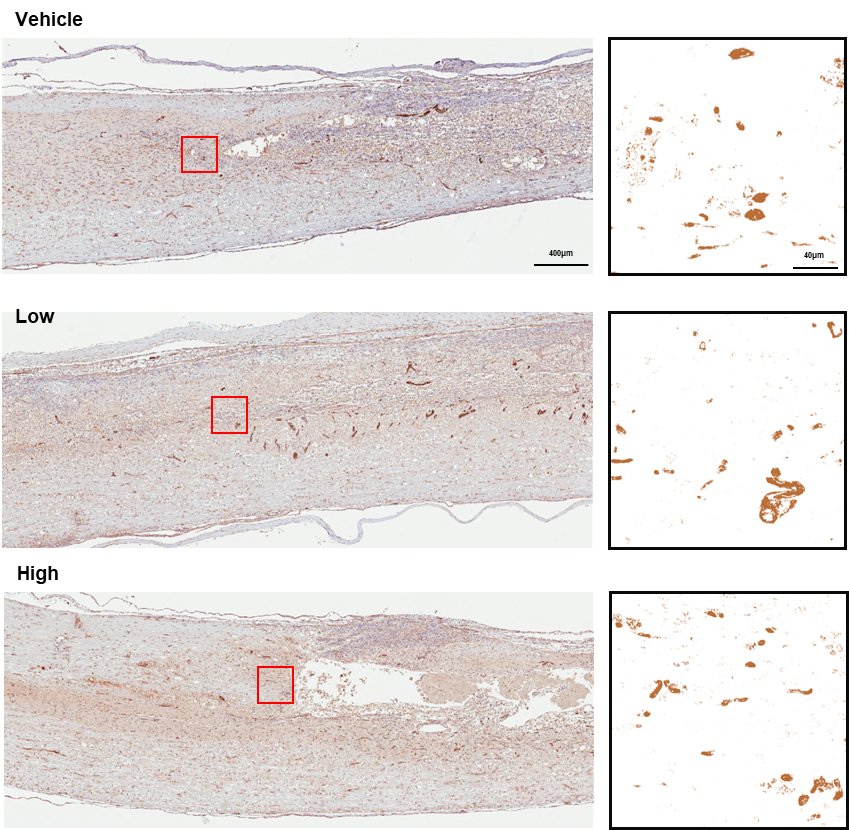

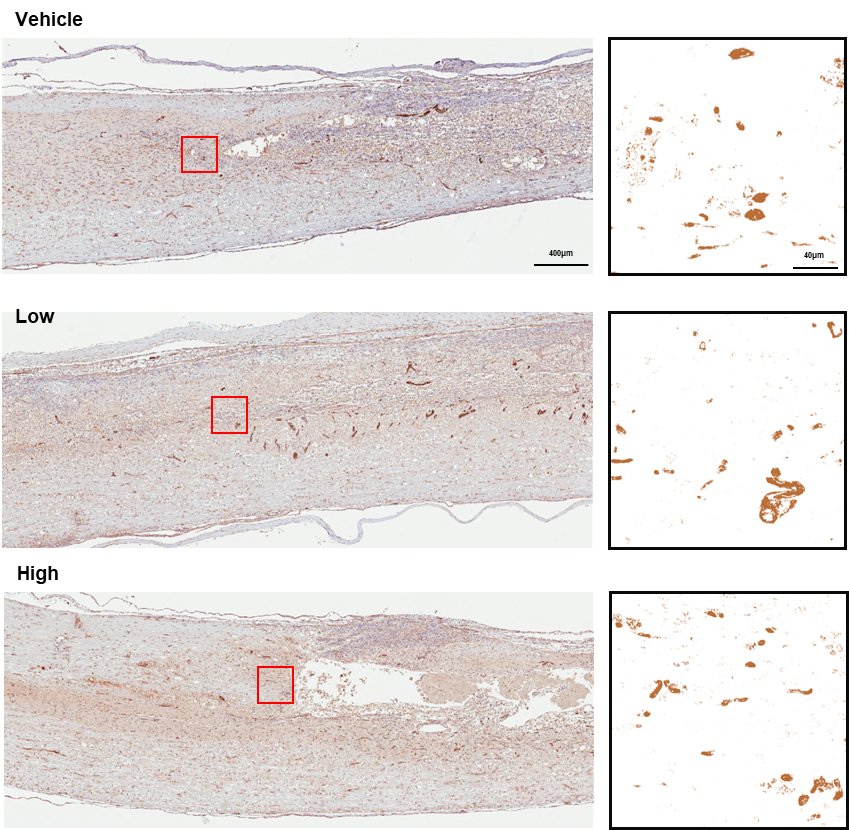

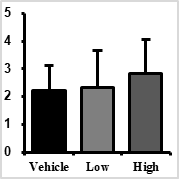


**Vessle area (%)**

**I**

**J**

**K**

**Vehicle**

**Low**

**High**

**A**

**B**

**Vehicle**

**Low**

**High**

**Vehicle**

**Low**

**High**

**Vehicle**

**Low**

**High**

**Iba 1**

**CD31**


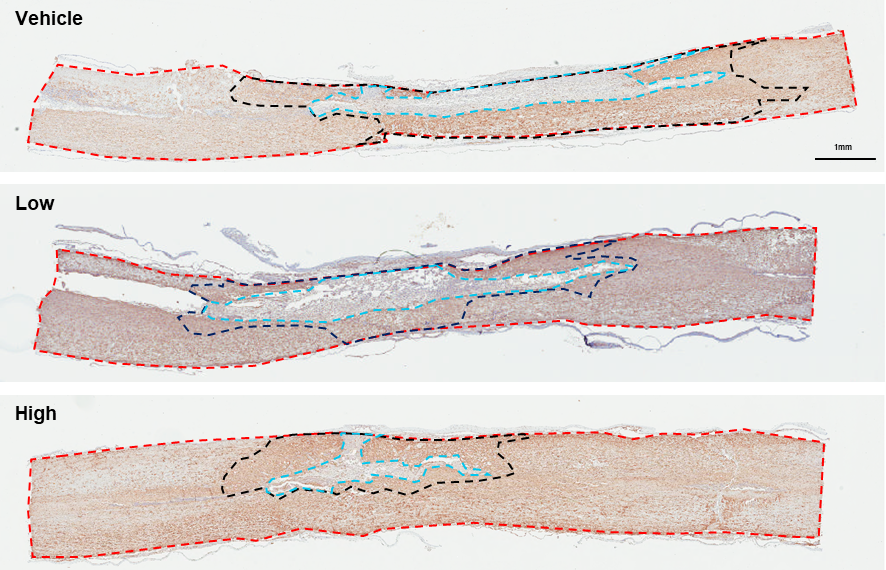

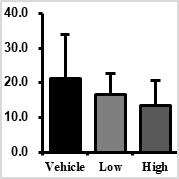


**

**Glial scar area (%)**

**Vehicle**

**Low**

**High**

**Red Box region**

**Red Box region**

**Red Box region**

**GFAP**

**Supplementary Figure 2. *In vivo* therapeutic effects of AhNSCs in SCI.**(**A, B**) Glial scar formation after SCI was examined by immunohistochemistry (IHC) against GFAP, a specific marker of astrocytes, at 6 weeks post-transplantation (WPT) of AhNSCs. Black and blue lines delineate the outer and inner contours of the glial scar, respectively. The average glial scar areas were calculated and compared. (**C, D, E**) Regeneration of axons and dendrites was analyzed by IHC against Tuj1, a specific marker of neurons, in the epicenter areas of SCI (red boxes). The staining intensities were analyzed and compared. (**F, G, H**) Neuroinflammation was analyzed by IHC against Iba1, a specific marker of microglia, in the penumbra areas of SCI (red boxes). The staining intensities were analyzed and compared. (**I, J, K**) Angiogenesis was analyzed by IHC against CD31, a specific marker of endothelial cells, in the penumbra areas of SCI (red boxes). The staining intensities were analyzed and compared. (**D, G, J**) Images represent magnified views of the SCI penumbra areas (red boxes), showing brown immunohistochemical staining results analyzed by ImageJ software after computational color deconvolution *, P < 0.05, **, P < 0.01.***, P < 0.001. compared with the vehicle group. Vehicle group (n = 37), Low-dose AhNSCs group (n = 37), High-dose AhNSCs group (n = 34).

**Supplementary Table 1. RT-PCR Validation for Detection of Residual AhNSCs**

| **Parameter** | **Details** |
| --- | --- |
| Methodology | Real-time PCR |
| Biological matrix | Sprague-Dawley rat genomic DNA |
| Analyte of interest | AhNSC |
| Specificity | No interference |
| Linearity | Calibration standards : 0.005 - 50 ng |
|  | Mean curve equation: y = -3.2365x+17.4921 (n=3) |
|  | Mean curve correlation : R^2^ = 0.997 (n=3) |
| Calibration curve range | 0.005 - 50 ng |
| QC levels | LQC : 0.015 ng |
|  | MQC : 0.5 ng |
|  | HQC : 40 ng |

**Supplementary Table 2. Body weight analysis in general toxicity**

| **Rat/Athymic nude rat Male (g)** | | | | | | **Female (g)** | | | |
| --- | --- | --- | --- | --- | --- | --- | --- | --- | --- |
|  |  | **Vehicle** | **Low** | **Medium** | **High** | **Vehicle** | **Low** | **Medium** | **High** |
|  |  | 0 | 1 × 10^5^ | 1 × 10^6^ | 3 × 10^6^ | 0 | 1 × 10^5^ | 1 × 10^6^ | 3 × 10^6^ |
| **Acute** | | | | | | | | | |
| **Day1** | (n) | 15 | 10 | 10 | 10 | 15 | 10 | 10 | 10 |
|  | Means | 174 | 177.2 | 178.1 | 174.4 | 162.1 | 164.6 | 166.3 | 165.2 |
|  | Sdevs | 30.95 | 22.53 | 25.31 | 30.27 | 13.1 | 18.73 | 13.94 | 13.6 |
| **Day8** | (n) | 15 | 10 | 10 | 10 | 15 | 10 | 10 | 10 |
|  | Means | 219.7 | 221.5 | 223.9 | 214.9 | 176.9 | 180.3 | 181 | 180.7 |
|  | Sdevs | 27.99 | 21.24 | 23.07 | 29.39 | 12.84 | 21.13 | 14.47 | 11.57 |
| **Day15** | (n) | 15 | 10 | 10 | 10 | 15 | 10 | 10 | 10 |
|  | Means | 256.4 | 262.3 | 263.6 | 252.9 | 187.3 | 194.2 | 193.9 | 190.9 |
|  | Sdevs | 22.19 | 21.45 | 21.14 | 26.45 | 12.68 | 20.13 | 15.96 | 10.84 |
| **Day22** | (n) | 15 | 10 | 10 | 10 | 15 | 10 | 10 | 10 |
|  | Means | 289.7 | 293.3 | 294.3 | 284.2 | 193.3 | 200 | 200.7 | 199.3 |
|  | Sdevs | 19.15 | 17.32 | 19.09 | 25.46 | 14.26 | 18.14 | 16.8 | 9.7 |
| **Day28** | (n) | 15 | 10 | 10 | 10 | 15 | 10 | 10 | 10 |
|  | Means | 305.7 | 308.5 | 284.2 | 298.8 | 203.3 | 208.7 | 210.3 | 207.7 |
|  | Sdevs | 22.05 | 17.93 | 25.46 | 25.6 | 12.43 | 20.52 | 19.01 | 12.85 |
| **Chronic** | | | | | | | | | |
| **Day1** | (n) | 5 | - | | 5 | 5 |  |  | 5 |
|  | Means | 304.1 |  |  | 276.1 | 204.7 |  |  | 212.4 |
|  | Sdevs | 20.35 |  |  | 18.41 | 15.22 |  |  | 14.36 |
| **Day8** | (n) | 5 |  |  | 5 | 5 |  |  | 5 |
|  | Means | 329.8 |  |  | **296.2*** | 217.5 |  |  | 224.3 |
|  | Sdevs | 23.26 |  |  | 18.33 | 11.75 |  |  | 11.05 |
| **Day15** | (n) | 5 |  |  | 5 | 5 |  |  | 5 |
|  | Means | 355.1 |  |  | **315.1*** | 222 |  |  | 229.4 |
|  | Sdevs | 26.88 |  |  | 19.18 | 15.1 |  |  | 14.74 |
| **Day22** | (n) | 5 |  |  | 5 | 5 |  |  | 5 |
|  | Means | 370.4 |  |  | **327.4*** | 219.5 |  |  | 230.3 |
|  | Sdevs | 26.56 |  |  | 21.29 | 14.65 |  |  | 12.2 |
| **Day29** | (n) | 5 |  |  | 5 | 5 |  |  | 5 |
|  | Means | 380.8 |  |  | **334.9*** | 223.4 |  |  | 236.7 |
|  | Sdevs | 31.43 |  |  | 24.14 | 15.57 |  |  | 11.9 |
| **Day36** | (n) | 5 |  |  | 5 | 5 |  |  | 5 |
|  | Means | 399 |  |  | **347.6*** | 227.1 |  |  | 242.9 |
|  | Sdevs | 32.84 |  |  | 24.2 | 27.16 |  |  | 12.16 |
| **Day43** | (n) | 5 |  |  | 5 | 5 |  |  | 5 |
|  | Means | 410.5 |  |  | **362.7*** | 228.1 |  |  | 242.4 |
|  | Sdevs | 33.26 |  |  | 24.69 | 24.6 |  |  | 14.32 |
| **Day50** | (n) | 5 |  |  | 5 | 5 |  |  | 5 |
|  | Means | 42.3 |  |  | **370.8*** | 233.5 |  |  | 243.4 |
|  | Sdevs | 36.46 |  |  | 27.19 | 26.13 |  |  | 12.34 |
| **Day57** | (n) | 5 |  |  | 5 | 5 |  |  | 5 |
|  | Means | 423.2 |  |  | **376.5*** | 233.5 |  |  | 233.5 |
|  | Sdevs | 32.48 |  |  | 27.55 | 26.37 |  |  | 26.37 |
| **Day63** | (n) | 5 |  |  | 5 | 5 |  |  | 5 |
|  | Means | 437.5 |  |  | **385.4*** | 244.8 |  |  | 248 |
|  | Sdevs | 36.41 |  |  | 24.33 | 13.85 |  |  | 14.34 |

*, P < 0.05

**Supplementary Table 3. Food consumption analysis in general toxicity study**

|  | |  | **Male (g/day)** | | | | **Female (g/day)** | | | |
| --- | --- | --- | --- | --- | --- | --- | --- | --- | --- | --- |
|  |  | | **Vehicle** | **Low** | **Medium** | **High** | **Vehicle** | **Low** | **Medium** | **High** |
|  |  |  | 0 | 1 × 10^5^ | 1 × 10^6^ | 3 × 10^6^ | 0 | 1 × 10^5^ | 1 × 10^6^ | 3 × 10^6^ |
| **Acute** | | | | | | | | | | |
| **Pre-Treatment**  **Day5** | (n) | | 15 | 10 | 10 | 15 | 15 | 10 | 10 | 15 |
|  | Means | | 18.0 | 17.9 | 18.1 | 17.8 | 15.4 | 15.4 | 15.5 | 15.2 |
|  | Sdevs | | 1.19 | 1.20 | 0.34 | 0.87 | 1.04 | 0.52 | 0.50 | 0.31 |
| **Treatment**  **Day8** | (n) | | 15 | 10 | 10 | 15 | 15 | 10 | 10 | 15 |
|  | Means | | 21.2 | 21.7 | 21.7 | 20.6 | 18.4 | 18.7 | **17.6*** | 18.5 |
|  | Sdevs | | 0.96 | 1.20 | 0.56 | 1.58 | 0.85 | 0.62 | 0.44 | 0.40 |
| **Day15** | (n) | | 15 | 10 | 10 | 10 | 15 | 10 | 10 | 10 |
|  | Means | | 23.6 | 24.5 | 24.5 | 23.4 | 18.5 | 18.9 | 18.4 | **14.9*** |
|  | Sdevs | | 1.25 | 1.00 | 1.27 | 1.26 | 1.04 | 0.75 | 1.12 | 3.28 |
| **Day22** | (n) | | 15 | 10 | 10 | 15 | 15 | 10 | 10 | 15 |
|  | Means | | 25.1 | 25.4 | 25.4 | 22.2 | 18.6 | 18.6 | 18.5 | 18.8 |
|  | Sdevs | | 1.81 | 0.47 | 0.85 | 4.30 | 0.83 | 0.67 | 0.61 | 0.48 |
| **Day28** | (n) | | 15 | 10 | 10 | 15 | 15 | 10 | 10 | 15 |
|  | Means | | 24.5 | 24.5 | 24.3 | 24.5 | 18.8 | 18.7 | 18.7 | 18.9 |
|  | Sdevs | | 1.66 | 0.56 | 1.20 | 2.19 | 0.89 | 0.80 | 1.37 | 1.48 |
| **Chronic** | | | | | | | | | | |
| **Day8** | (n) | | 5 |  | | 5 | 5 |  |  | 5 |
|  | Means | | 25.0 |  |  | **22.9*** | 20.0 |  |  | 20.6 |
|  | Sdevs | | 0.52 |  |  | 1.18 | 0.74 |  |  | 0.31 |
| **Day15** | (n) | | 5 |  |  | 5 | 5 |  |  | 5 |
|  | Means | | 25.0 |  |  | **23.5*** | 18.7 |  |  | 20.2 |
|  | Sdevs | | 0.26 |  |  | 0.85 | 1.90 |  |  | 0.82 |
| **Day22** | (n) | | 5 |  |  | 5 | 5 |  |  | 5 |
|  | Means | | 26.5 |  |  | **24.0*** | 17.9 |  |  | 18.9 |
|  | Sdevs | | 0.43 |  |  | 0.5 | 0.29 |  |  | 1.22 |
| **Day29** | (n) | | 5 |  |  | 5 | 5 |  |  | 5 |
|  | Means | | 25.0 |  |  | **22.7*** | 18.4 |  |  | 18.5 |
|  | Sdevs | | 0.00 |  |  | 0.27 | 0.15 |  |  | 1.48 |
| **Day36** | (n) | | 5 |  |  | 5 | 5 |  |  | 5 |
|  | Means | | 27.2 |  |  | **24.4*** | 19.4 |  |  | 19.4 |
|  | Sdevs | | 0.64 |  |  | 0.24 | 0.31 |  |  | 1.08 |
| **Day43** | (n) | | 2 |  |  | 5 | 5 |  |  | 5 |
|  | Means | | 26.4 |  |  | **24.6*** | 18.9 |  |  | 19.3 |
|  | Sdevs | | 0.51 |  |  | 0.37 | 0.38 |  |  | 0.78 |
| **Day50** | (n) | | 5 |  |  | 5 | 5 |  |  | 5 |
|  | Means | | 27.1 |  |  | **24.4*** | 20.7 |  |  | **19.3*** |
|  | Sdevs | | 0.75 |  |  | 0.4 | 0.86 |  |  | 0.58 |
| **Day57** | (n) | | 5 |  |  | 5 | 5 |  |  | 5 |
|  | Means | | 24.1 |  |  | 23.6 | 18.4 |  |  | 18.7 |
|  | Sdevs | | 6.83 |  |  | 4.46 | 0.76 |  |  | 0.89 |
| **Day63** | (n) | | 5 |  |  | 5 | 5 |  |  | 5 |
|  | Means | | 26.9 |  |  | **23.8*** | 18.7 |  |  | 18.5 |
|  | Sdevs | | 0.18 |  |  | 0.33 | 1.17 |  |  | 0.69 |

*, P < 0.05

**Supplementary Table 4. Hematology analysis in general toxicity study (Male)**

*, P < 0.05

| **Rat/Athymic nude rat** | | **Male** | | | | | | |
| --- | --- | --- | --- | --- | --- | --- | --- | --- |
|  |  | **Day29** | | | |  | **Day64** | |
|  |  | **Vehicle** | **Low** | **Medium** | **High** |  | **Vehicle** | **High** |
|  |  | 0 | 1 × 10^5^ | 1 × 10^6^ | 3 × 10^6^ |  | 0 | 3 × 10^6^ |
| **RBC** (10^6^/μL) | (n) | 10 | 10 | 10 | 10 | (n) | 5 | 5 |
|  | Means | 8.80 | 8.70 | 8.70 | 8.54 | Means | 9.13 | 9.28 |
|  | Sdevs | 0.198 | 0.227 | 0.489 | 0.651 | Sdevs | 0.331 | 0.310 |
| **HGB** (g/dL) | (n) | 10 | 10 | 10 | 10 | (n) | 5 | 5 |
|  | Means | 16.2 | 16.2 | 16.1 | 15.8 | Means | 16.1 | 16.4 |
|  | Sdevs | 0.41 | 0.41 | 0.74 | 1.00 | Sdevs | 0.48 | 0.43 |
| **HCT** (%) | (n) | 10 | 10 | 10 | 10 | (n) | 5 | 5 |
|  | Means | 50.6 | 50.5 | 50.3 | 49.6 | Means | 50.9 | 51.7 |
|  | Sdevs | 1.28 | 1.58 | 2.40 | 2.81 | Sdevs | 2.04 | 1.85 |
| **MCV** (fL) | (n) | 10 | 10 | 10 | 10 | (n) | 5 | 5 |
|  | Means | 57.5 | 58.1 | 57.8 | 58.2 | Means | 55.7 | 55.7 |
|  | Sdevs | 1.04 | 1.11 | 1.17 | 1.93 | Sdevs | 0.57 | 0.59 |
| **MCH** (pg) | (n) | 10 | 10 | 10 | 10 | (n) | 5 | 5 |
|  | Means | 18.4 | 18.6 | 18.5 | 18.5 | Means | 17.7 | 17.6 |
|  | Sdevs | 0.35 | 0.43 | 0.35 | 0.60 | Sdevs | 0.50 | 0.19 |
| **MCHC** (g/dL) | (n) | 10 | 10 | 10 | 10 | (n) | 5 | 5 |
|  | Means | 32.1 | 32.1 | 32.1 | 31.8 | Means | 31.8 | 31.7 |
|  | Sdevs | 0.58 | 0.66 | 0.79 | 0.71 | Sdevs | 0.69 | 0.58 |
| **RET%** (%) | (n) | 10 | 10 | 10 | 10 | (n) | 5 | 5 |
|  | Means | 2.73 | 2.60 | 2.97 | 3.69 | Means | 2.51 | 2.52 |
|  | Sdevs | 0.360 | 0.403 | 0.795 | 3.007 | Sdevs | 0.111 | 0.406 |
| **RETA** (10^9^/L) | (n) | 10 | 10 | 10 | 10 | (n) | 5 | 5 |
|  | Means | 241 | 225 | 257 | 299 | Means | 229 | 234 |
|  | Sdevs | 35.3 | 30.7 | 60.1 | 191.6 | Sdevs | 15.9 | 41.3 |
| **PLT** (10^3^/uL) | (n) | 10 | 10 | 10 | 10 | (n) | 5 | 5 |
|  | Means | 956.8 | 945.2 | 972.9 | 992.2 | Means | 950.2 | 949.0 |
|  | Sdevs | 87.00 | 83.52 | 116.37 | 212.23 | Sdevs | 65.07 | 40.54 |
| **NEU%** (%) | (n) | 10 | 10 | 10 | 10 | (n) | 5 | 5 |
|  | Means | 45.1 | 40.9 | 42.6 | 46.8 | Means | 52.7 | 50.4 |
|  | Sdevs | 5.66 | 9.39 | 6.74 | 7.28 | Sdevs | 13.05 | 8.59 |
| **LYM%** (%) | (n) | 10 | 10 | 10 | 10 | (n) | 5 | 5 |
|  | Means | 44.8 | 48.7 | 46.9 | 43.1 | Means | 39.4 | 41.5 |
|  | Sdevs | 5.00 | 9.85 | 6.43 | 6.48 | Sdevs | 13.14 | 8.56 |
| **EOS%** (%) | (n) | 10 | 10 | 10 | 10 | (n) | 5 | 5 |
|  | Means | 1.6 | 1.8 | 2.1 | 1.5 | Means | 2.3 | 1.9 |
|  | Sdevs | 0.59 | 0.55 | 1.62 | 0.39 | Sdevs | 0.64 | 0.28 |
| **MON%** (%) | (n) | 10 | 10 | 10 | 10 | (n) | 5 | 5 |
|  | Means | 7.7 | 7.9 | 7.4 | 7.8 | Means | 5.1 | 5.7 |
|  | Sdevs | 1.41 | 1.36 | 1.55 | 1.25 | Sdevs | 0.88 | 0.27 |
| **BAS%** (%) | (n) | 10 | 10 | 10 | 10 | (n) | 5 | 5 |
|  | Means | 0.4 | 0.5 | 0.5 | 0.5 | Means | 0.3 | 0.3 |
|  | Sdevs | 0.17 | 0.12 | 0.26 | 0.12 | Sdevs | 0.11 | 0.11 |
| **LUC%** (%) | (n) | 10 | 10 | 10 | 10 | (n) | 5 | 5 |
|  | Means | 0.4 | 0.4 | 0.4 | 0.4 | Means | 0.2 | 0.2 |
|  | Sdevs | 0.15 | 0.11 | 0.11 | 0.12 | Sdevs | 0.07 | 0.07 |
| **WBC** (%) | (n) | 10 | 10 | 10 | 10 | (n) | 5 | 5 |
|  | Means | 6.47 | 6.26 | 7.26 | 6.83 | Means | 9.26 | 9.73 |
|  | Sdevs | 1.264 | 1.329 | 1.254 | 1.882 | Sdevs | 0.464 | 2.973 |
| **NEUA** (10^3^/uL) | (n) | 10 | 10 | 10 | 10 | (n) | 5 | 5 |
|  | Means | 2.91 | 2.56 | 3.15 | 3.28 | Means | 4.84 | 4.93 |
|  | Sdevs | 0.625 | 0.909 | 0.938 | 1.426 | Sdevs | 0.973 | 1.648 |
| **LYMA** (10^3^/uL) | (n) | 10 | 10 | 10 | 10 | (n) | 5 | 5 |
|  | Means | 2.92 | 3.05 | 3.35 | 2.86 | Means | 3.69 | 4.02 |
|  | Sdevs | 0.704 | 0.882 | 0.370 | 0.443 | Sdevs | 1.351 | 1.463 |
| **MONA** (10^3^/uL) | (n) | 10 | 10 | 10 | 10 | (n) | 5 | 5 |
|  | Means | 0.50 | 0.49 | 0.55 | 0.53 | Means | 0.47 | 0.55 |
|  | Sdevs | 0.135 | 0.100 | 0.164 | 0.135 | Sdevs | 0.091 | 0.188 |
| **EOSA** (10^3^/uL) | (n) | 10 | 10 | 10 | 10 | (n) | 5 | 5 |
|  | Means | 0.10 | 0.11 | 0.16 | 0.10 | Means | 0.21 | 0.18 |
|  | Sdevs | 0.033 | 0.037 | 0.118 | 0.033 | Sdevs | 0.062 | 0.043 |
| **BASA** (10^3^/uL) | (n) | 10 | 10 | 10 | 10 | (n) | 5 | 5 |
|  | Means | 0.03 | 0.03 | 0.03 | 0.04 | Means | 0.03 | 0.04 |
|  | Sdevs | 0.012 | 0.009 | 0.019 | 0.011 | Sdevs | 0.011 | 0.009 |
| **LUCA** (10^3^/uL) | (n) | 10 | 10 | 10 | 10 | (n) | 5 | 5 |
|  | Means | 0.02 | 0.02 | 0.03 | 0.03 | Means | 0.02 | 0.02 |
|  | Sdevs | 0.011 | 0.008 | 0.009 | 0.011 | Sdevs | 0.007 | 0.007 |
| **PT** (sec) | (sec) | 10 | 10 | 10 | 10 | (n) | 10 | 10 |
|  | Means | 10.6 | 10.6 | 10.9 | **11.3*** | Means | 13.2 | 12.8 |
|  | Sdevs | 0.57 | 0.27 | 0.55 | 0.75 | Sdevs | 0.65 | 0.64 |
| **APTT** (10^3^/uL) | (n) | 10 | 10 | 10 | 10 | (n) | 10 | 10 |
|  | Means | 13.1 | 13.7 | 13.5 | 12.3 | Means | 18.9 | 17.7 |
|  | Sdevs | 2.40 | 1.85 | 1.49 | 1.69 | Sdevs | 1.34 | 2.35 |

**Supplemengary Table 4. Hematology analysis in general toxicity study (Female)**

| **Rat/Athymic nude rat** | | **Female** | | | | | | |
| --- | --- | --- | --- | --- | --- | --- | --- | --- |
|  |  | **Day29** | | | |  | **Day64** | |
|  |  | **Vehicle** | **Low** | **Medium** | **High** |  | **Vehicle** | **High** |
|  |  | 0 | 1 × 10^5^ | 1 × 10^6^ | 3 × 10^6^ |  | 0 | 3 × 10^6^ |
| **RBC** (10^6^/μL) | (n) | 10 | 10 | 10 | 10 | (n) | 5 | 5 |
|  | Means | 8.93 | 9.01 | **9.39*** | 9.12 | Means | 8.21 | 9.02 |
|  | Sdevs | 0.358 | 0.416 | 0.361 | 0.300 | Sdevs | 1.654 | 0.343 |
| **HGB** (g/dL) | (n) | 10 | 10 | 10 | 10 | (n) | 5 | 5 |
|  | Means | 16.1 | 16.3 | 16.4 | 16.4 | Means | 13.8 | 15.8 |
|  | Sdevs | 0.55 | 0.77 | 0.64 | 0.64 | Sdevs | 4.09 | 0.59 |
| **HCT** (%) | (n) | 10 | 10 | 10 | 10 | (n) | 5 | 5 |
|  | Means | 50.2 | 50.9 | 51.2 | 51.2 | Means | 45.3 | 51.1 |
|  | Sdevs | 1.94 | 1.84 | 1.82 | 1.82 | Sdevs | 11.00 | 1.67 |
| **MCV** (fL) | (n) | 10 | 10 | 10 | 10 | (n) | 5 | 5 |
|  | Means | 56.2 | 56.5 | 56.1 | 56.1 | Means | 54.7 | 56.6 |
|  | Sdevs | 0.44 | 0.76 | 0.69 | 0.69 | Sdevs | 3.35 | 0.34 |
| **MCH** (pg) | (n) | 10 | 10 | 10 | 10 | (n) | 5 | 5 |
|  | Means | 18.0 | 18.1 | 18.0 | 18.0 | Means | 16.4 | 17.6 |
|  | Sdevs | 0.23 | 0.20 | 0.29 | 0.29 | Sdevs | 2.33 | 0.11 |
| **MCHC** (g/dL) | (n) | 10 | 10 | 10 | 10 | (n) | 5 | 5 |
|  | Means | 32.1 | 32.0 | 32.2 | 32.2 | Means | 29.9 | 31.0 |
|  | Sdevs | 0.33 | 0.49 | 0.44 | 0.44 | Sdevs | 2.61 | 0.20 |
| **RET%** (%) | (n) | 10 | 10 | 10 | 10 | (n) | 5 | 5 |
|  | Means | 3.16 | 2.91 | 2.92 | 2.92 | Means | 5.37 | 2.71 |
|  | Sdevs | 0.403 | 0.651 | 0.410 | 0.410 | Sdevs | 6.162 | 0.355 |
| **RETA** (10^9^/L) | (n) | 10 | 10 | 10 | 10 | (n) | 5 | 5 |
|  | Means | 283 | 262 | 266 | 266 | Means | 360 | 244 |
|  | Sdevs | 38.7 | 55.6 | 39.7 | 39.7 | Sdevs | 285.5 | 25.1 |
| **PLT** (10^3^/uL) | (n) | 10 | 10 | 10 | 10 | (n) | 5 | 5 |
|  | Means | 970.0 | 1006.4 | 950.1 | 950.1 | Means | 1290.6 | 982.0 |
|  | Sdevs | 70.49 | 77.19 | 110.95 | 110.95 | Sdevs | 731.54 | 76.87 |
| **NEU%** (%) | (n) | 10 | 10 | 10 | 10 | (n) | 5 | 5 |
|  | Means | 38.7 | 36.5 | 34.9 | 38.6 | Means | 45.5 | 43.0 |
|  | Sdevs | 5.30 | 6.90 | 6.81 | 17.39 | Sdevs | 22.96 | 6.26 |
| **LYM%** (%) | (n) | 10 | 10 | 10 | 10 | (n) | 5 | 5 |
|  | Means | 49.7 | 52.0 | 54.0 | 50.2 | Means | 45.2 | 46.6 |
|  | Sdevs | 5.42 | 7.68 | 6.68 | 15.04 | Sdevs | 22.03 | 7.68 |
| **EOS%** (%) | (n) | 10 | 10 | 10 | 10 | (n) | 5 | 5 |
|  | Means | 3.7 | 3.4 | 3.3 | 3.0 | Means | 3.4 | 3.6 |
|  | Sdevs | 1.21 | 1.30 | 1.31 | 1.31 | Sdevs | 1.38 | 1.81 |
| **MON%** (%) | (n) | 10 | 10 | 10 | 10 | (n) | 5 | 5 |
|  | Means | 6.8 | 7.0 | 6.9 | 7.2 | Means | 4.9 | 5.9 |
|  | Sdevs | 1.85 | 2.01 | 1.03 | 2.11 | Sdevs | 2.05 | 1.68 |
| **BAS%** (%) | (n) | 10 | 10 | 10 | 10 | (n) | 5 | 5 |
|  | Means | 0.5 | 0.6 | 0.5 | 0.5 | Means | 0.6 | 0.5 |
|  | Sdevs | 0.29 | 0.22 | 0.25 | 0.28 | Sdevs | 0.39 | 0.16 |
| **LUC%** (%) | (n) | 10 | 10 | 10 | 10 | (n) | 5 | 5 |
|  | Means | 0.5 | 0.5 | 0.4 | 0.4 | Means | 0.4 | 0.5 |
|  | Sdevs | 0.23 | 0.13 | 0.20 | 0.29 | Sdevs | 0.19 | 0.25 |
| **WBC** (%) | (n) | 10 | 10 | 10 | 10 | (n) | 5 | 5 |
|  | Means | 4.38 | 5.05 | 4.95 | 5.93 | Means | 20.51 | 3.93 |
|  | Sdevs | 1.270 | 1.723 | 1.216 | 1.739 | Sdevs | 37.533 | 0.783 |
| **NEUA** (10^3^/uL) | (n) | 10 | 10 | 10 | 10 | (n) | 5 | 5 |
|  | Means | 1.74 | 1.82 | 1.74 | 2.40 | Means | 16.12 | 1.71 |
|  | Sdevs | 0.685 | 0.573 | 0.556 | 1.507 | Sdevs | 33.129 | 0.530 |
| **LYMA** (10^3^/uL) | (n) | 10 | 10 | 10 | 10 | (n) | 5 | 5 |
|  | Means | 2.13 | 2.66 | 2.65 | 2.89 | Means | 2.82 | 1.81 |
|  | Sdevs | 0.460 | 1.246 | 0.653 | 0.999 | Sdevs | 1.748 | 0.362 |
| **MONA** (10^3^/uL) | (n) | 10 | 10 | 10 | 10 | (n) | 5 | 5 |
|  | Means | 0.31 | 0.36 | 0.35 | 0.42 | Means | 0.56 | 0.23 |
|  | Sdevs | 0.148 | 0.142 | 0.114 | 0.159 | Sdevs | 0.786 | 0.059 |
| **EOSA** (10^3^/uL) | (n) | 10 | 10 | 10 | 10 | (n) | 5 | 5 |
|  | Means | 0.16 | 0.17 | 0.16 | 0.17 | Means | 0.88 | 0.14 |
|  | Sdevs | 0.045 | 0.066 | 0.086 | 0.078 | Sdevs | 1.722 | 0.077 |
| **BASA** (10^3^/uL) | (n) | 10 | 10 | 10 | 10 | (n) | 5 | 5 |
|  | Means | 0.02 | 0.03 | 0.02 | 0.03 | Means | 0.07 | 0.02 |
|  | Sdevs | 0.012 | 0.009 | 0.008 | 0.017 | Sdevs | 0.105 | 0.008 |
| **LUCA** (10^3^/uL) | (n) | 10 | 10 | 10 | 10 | (n) | 5 | 5 |
|  | Means | 0.02 | 0.02 | 0.02 | 0.03 | Means | 0.05 | 0.02 |
|  | Sdevs | 0.010 | 0.007 | 0.009 | 0.016 | Sdevs | 0.083 | 0.008 |
| **PT** (sec) | (sec) | 10 | 10 | 10 | 10 | (n) | 10 | 10 |
|  | Means | 10.6 | 10.6 | 10.9 | **11.3*** | Means | 10.6 | 10.9 |
|  | Sdevs | 0.57 | 0.27 | 0.55 | 0.75 | Sdevs | 0.64 | 0.65 |
| **APTT** (10^3^/uL) | (n) | 10 | 10 | 10 | 10 | (n) | 10 | 10 |
|  | Means | 13.1 | 13.7 | 13.5 | 12.3 | Means | 15.2 | 16.5 |
|  | Sdevs | 2.40 | 1.85 | 1.49 | 1.69 | Sdevs | 1.18 | 0.89 |

*, P < 0.05

**Supplementary Table 5. Clinical chemistry analysis in general toxicity study (Male)**

| **Rat/Athymic nude rat** | | **Male** | | | | | | |
| --- | --- | --- | --- | --- | --- | --- | --- | --- |
|  |  | **Day29** | | | |  | **Day64** | |
|  |  | **Vehicle** | **Low** | **Medium** | **High** |  | **Vehicle** | **High** |
|  |  | 0 | 1 × 10^5^ | 1 × 10^6^ | 3 × 10^6^ |  | 0 | 3 × 10^6^ |
| **GLU** (mg/dL) | (n) | 10 | 10 | 10 | 10 | (n) | 10 | 10 |
|  | Means | 96.8 | 88.8 | 87.1 | 86.9 | Means | 110.6 | 104.3 |
|  | Sdevs | 30.81 | 18.96 | 20.89 | 18.93 | Sdevs | 29.48 | 35.43 |
| **BUN** (mg/dL) | (n) | 10 | 10 | 10 | 10 | (n) | 10 | 10 |
|  | Means | 19.3 | 19.6 | 20.6 | 20.8 | Means | 18.2 | 19.8 |
|  | Sdevs | 3.06 | 3.15 | 3.44 | 2.67 | Sdevs | 2.64 | 2.23 |
| **CREA** (mg/dL) | (n) | 10 | 10 | 10 | 10 | (n) | 10 | 10 |
|  | Means | 0.38 | 0.39 | 0.36 | 0.38 | Means | 0.43 | 0.42 |
|  | Sdevs | 0.034 | 0.038 | 0.021 | 0.038 | Sdevs | 0.04 | 0.028 |
| **TP** (g/dL) | (n) | 10 | 10 | 10 | 10 | (n) | 10 | 10 |
|  | Means | 5.85 | 6.06 | 5.89 | 5.95 | Means | 6.02 | 6.03 |
|  | Sdevs | 0.287 | 0.351 | 0.284 | 0.259 | Sdevs | 0.230 | 0.240 |
| **ALB** (g/dL) | (n) | 10 | 10 | 10 | 10 | (n) | 10 | 10 |
|  | Means | 4.44 | 4.51 | 4.36 | 4.35 | Means | 4.11 | 4.31 |
|  | Sdevs | 0.223 | 0.274 | 0.170 | 0.470 | Sdevs | 0.081 | 0.228 |
| **A/G** (ratio) | (n) | 10 | 10 | 10 | 10 | (n) | 10 | 10 |
|  | Means | 3.19 | 2.93 | 2.86 | 2.84 | Means | 2.18 | 2.55 |
|  | Sdevs | 0.373 | 0.231 | 0.248 | 0.591 | Sdevs | 0.291 | 0.376 |
| **AST** (IU/L) | (n) | 10 | 10 | 10 | 10 | (n) | 10 | 10 |
|  | Means | 93.0 | 89.9 | 96.8 | 97.7 | Means | 101.8 | 103.9 |
|  | Sdevs | 9.30 | 9.25 | 9.34 | 11.16 | Sdevs | 11.54 | 5.34 |
| **ALT** (IU/L) | (n) | 10 | 10 | 10 | 10 | (n) | 10 | 10 |
|  | Means | 21.1 | 20.2 | 22.1 | 22.0 | Means | 26.7 | 25.6 |
|  | Sdevs | 2.29 | 2.82 | 3.05 | 2.51 | Sdevs | 5.16 | 3.62 |
| **TBIL** (mg/dL) | (n) | 10 | 10 | 10 | 10 | (n) | 10 | 10 |
|  | Means | 0.108 | 0.111 | 0.107 | 0.111 | Means | 0.113 | 0.120 |
|  | Sdevs | 0.0081 | 0.0061 | 0.0079 | 0.0081 | Sdevs | 0.0040 | 0.0081 |
| **GGT** (IU/L) | (n) | 10 | 10 | 10 | 10 | (n) | 10 | 10 |
|  | Means | 0.89 | 0.76 | 0.93 | 1.03 | Means | 0.51 | 0.46 |
|  | Sdevs | 0.446 | 0.256 | 0.778 | 0.752 | Sdevs | 0.271 | 0.375 |
| **ALP** (IU/L) | (n) | 10 | 10 | 10 | 10 | (n) | 10 | 10 |
|  | Means | 300.5 | 327.1 | 340.6 | **365.8*** | Means | 245.1 | 243.4 |
|  | Sdevs | 54.04 | 39.24 | 47.16 | 39.66 | Sdevs | 44.80 | 31.13 |
| **TCHO** (mg/dL) | (n) | 10 | 10 | 10 | 10 | (n) | 10 | 10 |
|  | Means | 77.3 | 76.0 | 69.3 | 71.6 | Means | 68.8 | 67.8 |
|  | Sdevs | 12.78 | 14.21 | 7.41 | 8.62 | Sdevs | 8.32 | 8.04 |
| **TG** (mg/dL) | (n) | 10 | 10 | 10 | 10 | (n) | 10 | 10 |
|  | Means | 14.9 | 13.2 | 10.7 | 13.9 | Means | 12.0 | 10.8 |
|  | Sdevs | 6.73 | 3.70 | 2.68 | 5.58 | Sdevs | 3.37 | 2.62 |
| **Ca** (mg/dL) | (n) | 10 | 10 | 10 | 10 | (n) | 10 | 10 |
|  | Means | 10.67 | 10.97 | 10.81 | 10.83 | Means | 10.01 | 10.20 |
|  | Sdevs | 0.460 | 0.440 | 0.433 | 0.368 | Sdevs | 0.486 | 0.329 |
| **IP** (mg/dL) | (n) | 10 | 10 | 10 | 10 | (n) | 10 | 10 |
|  | Means | 10.89 | 10.97 | 11.09 | 11.12 | Means | 9.32 | 8.77 |
|  | Sdevs | 0.866 | 0.912 | 1.070 | 0.893 | Sdevs | 1.098 | 0.484 |
| **K** (mmol/L) | (n) | 10 | 10 | 10 | 10 | (n) | 10 | 10 |
|  | Means | 7.63 | 7.70 | 7.60 | 7.83 | Means | 8.05 | 7.56 |
|  | Sdevs | 0.649 | 0.681 | 0.684 | 0.951 | Sdevs | 1.329 | 1.156 |
| **CK** (IU/L) | (n) | 10 | 10 | 10 | 10 | (n) | 10 | 10 |
|  | Means | 481.7 | 445.9 | 473.2 | 469.5 | Means | 522.2 | 511.6 |
|  | Sdevs | 87.56 | 90.42 | 83.02 | 92.27 | Sdevs | 42.63 | 70.49 |
| **PL** (mg/dL) | (n) | 10 | 10 | 10 | 10 | (n) | 10 | 10 |
|  | Means | 102.0 | 99.6 | 91.3 | 97.1 | Means | 91.4 | 93.4 |
|  | Sdevs | 14.45 | 18.28 | 7.51 | 11.14 | Sdevs | 8.32 | 10.43 |
| **Na** (mmol/L) | (n) | 10 | 10 | 10 | 10 | (n) | 10 | 10 |
|  | Means | 146.7 | 147.8 | 147.4 | 148.0 | Means | 145.4 | 146.0 |
|  | Sdevs | 1.89 | 2.30 | 1.35 | 2.00 | Sdevs | 1.52 | 1.22 |
| **Cl** (mmol/L) | (n) | 10 | 10 | 10 | 10 | (n) | 10 | 10 |
|  | Means | 101.8 | 102.7 | 102.7 | 102.9 | Means | 99.4 | **101.2*** |
|  | Sdevs | 2.35 | 1.16 | 1.49 | 1.45 | Sdevs | 0.89 | 1.30 |

*, P < 0.05

**Supplementary Table 5. Clinical chemistry analysis in general toxicity study (Famale)**

| **Rat/Athymic nude rat** | | **Female** | | | | | | |
| --- | --- | --- | --- | --- | --- | --- | --- | --- |
|  |  | **Day29** | | | |  | **Day64** | |
|  |  | **Vehicle** | **Low** | **Medium** | **High** |  | **Vehicle** | **High** |
|  |  | 0 | 1 × 10^5^ | 1 × 10^6^ | 3 × 10^6^ |  | 0 | 3 × 10^6^ |
| **GLU** (mg/dL) | (n) | 10 | 10 | 10 | 10 | (n) | 10 | 10 |
|  | Means | 66.8 | 76.1 | 63.8 | 64.9 | Means | 93.8 | 81.6 |
|  | Sdevs | 11.10 | 27.32 | 8.60 | 10.40 | Sdevs | 21.08 | 14.35 |
| **BUN** (mg/dL) | (n) | 10 | 10 | 10 | 10 | (n) | 10 | 10 |
|  | Means | 28.0 | 28.0 | 29.2 | 29.3 | Means | 21.8 | 20.0 |
|  | Sdevs | 2.69 | 1.93 | 3.40 | 6.90 | Sdevs | 10.34 | 2.13 |
| **CREA** (mg/dL) | (n) | 10 | 10 | 10 | 10 | (n) | 10 | 10 |
|  | Means | 0.33 | 0.35 | 0.34 | 0.32 | Means | 0.34 | 0.38 |
|  | Sdevs | 0.032 | 0.039 | 0.027 | 0.041 | Sdevs | 0.092 | 0.014 |
| **TP** (g/dL) | (n) | 10 | 10 | 10 | 10 | (n) | 10 | 10 |
|  | Means | 5.84 | 6.13 | 6.03 | 5.83 | Means | 5.72 | 5.94 |
|  | Sdevs | 0.475 | 0.420 | 0.459 | 0.394 | Sdevs | 0.971 | 0.330 |
| **ALB** (g/dL) | (n) | 10 | 10 | 10 | 10 | (n) | 10 | 10 |
|  | Means | 4.45 | 4.70 | 4.56 | 4.42 | Means | 3.97 | 4.38 |
|  | Sdevs | 0.472 | 0.354 | 0.388 | 0.295 | Sdevs | 1.167 | 0.287 |
| **A/G** (ratio) | (n) | 10 | 10 | 10 | 10 | (n) | 10 | 10 |
|  | Means | 3.29 | 3.35 | 3.15 | 3.12 | Means | 2.38 | 2.81 |
|  | Sdevs | 0.577 | 0.449 | 0.518 | 0.175 | Sdevs | 0.873 | 0.297 |
| **AST** (IU/L) | (n) | 10 | 10 | 10 | 10 | (n) | 10 | 10 |
|  | Means | 99.4 | 92.3 | 97.4 | 112.1 | Means | 99.8 | 84.2 |
|  | Sdevs | 8.98 | 10.48 | 8.79 | 27.17 | Sdevs | 48.86 | 12.64 |
| **ALT** (IU/L) | (n) | 10 | 10 | 10 | 10 | (n) | 10 | 10 |
|  | Means | 21.1 | 21.2 | 21.6 | 23.9 | Means | 25.0 | 22.6 |
|  | Sdevs | 2.97 | 4.12 | 5.47 | 6.68 | Sdevs | 6.85 | 4.05 |
| **TBIL** (mg/dL) | (n) | 10 | 10 | 10 | 10 | (n) | 10 | 10 |
|  | Means | 9.126 | 0.127 | 0.127 | 0.123 | Means | 0.108 | **0.124*** |
|  | Sdevs | 0.0093 | 0.0112 | 0.0077 | 0.0080 | Sdevs | 0.0132 | 0.0083 |
| **GGT** (IU/L) | (n) | 10 | 10 | 10 | 10 | (n) | 10 | 10 |
|  | Means | 1.67 | 2.01 | 1.72 | 2.39 | Means | 5.06 | 1.58 |
|  | Sdevs | 0.412 | 1.063 | 0.683 | 0.913 | Sdevs | 9.142 | 0.560 |
| **ALP** (IU/L) | (n) | 10 | 10 | 10 | 10 | (n) | 10 | 10 |
|  | Means | 184.2 | 169.2 | 179.3 | 181.4 | Means | 668.4 | 108.1 |
|  | Sdevs | 30.05 | 41.03 | 20.88 | 39.53 | Sdevs | 1244.67 | 16.32 |
| **TCHO** (mg/dL) | (n) | 10 | 10 | 10 | 10 | (n) | 10 | 10 |
|  | Means | 83.3 | 82.8 | 83.5 | 79.8 | Means | 96.4 | 85.2 |
|  | Sdevs | 12.87 | 12.99 | 15.23 | 13.81 | Sdevs | 20.61 | 12.95 |
| **TG** (mg/dL) | (n) | 10 | 10 | 10 | 10 | (n) | 10 | 10 |
|  | Means | 13.6 | 14.6 | 10.9 | 11.6 | Means | 16.9 | 12.0 |
|  | Sdevs | 5.39 | 6.55 | 2.40 | 3.66 | Sdevs | 6.20 | 3.02 |
| **Ca** (mg/dL) | (n) | 10 | 10 | 10 | 10 | (n) | 10 | 10 |
|  | Means | 10.38 | 10.63 | 10.53 | 10.50 | Means | 9.69 | 9.76 |
|  | Sdevs | 0.559 | 0.415 | 0.418 | 0.660 | Sdevs | 0.576 | 0.256 |
| **IP** (mg/dL) | (n) | 10 | 10 | 10 | 10 | (n) | 10 | 10 |
|  | Means | 9.70 | 9.68 | 9.82 | 10.04 | Means | 8.49 | 7.82 |
|  | Sdevs | 0.738 | 1.049 | 0.496 | 0.920 | Sdevs | 2.030 | 0.342 |
| **K** (mmol/L) | (n) | 10 | 10 | 10 | 7 | (n) | 10 | 10 |
|  | Means | 7.01 | 6.95 | 6.83 | **7.71*** | Means | 7.44 | 7.16 |
|  | Sdevs | 0.507 | 0.550 | 0.494 | 0.546 | Sdevs | 0.862 | 0.371 |
| **CK** (IU/L) | (n) | 10 | 10 | 10 | 10 | (n) | 10 | 10 |
|  | Means | 612.5 | **506.3*** | 593.9 | 658.3 | Means | 400.8 | 373.0 |
|  | Sdevs | 77.19 | 72.43 | 68.27 | 192.86 | Sdevs | 122.63 | 61.62 |
| **PL** (mg/dL) | (n) | 10 | 10 | 10 | 10 | (n) | 10 | 10 |
|  | Means | 123.0 | 124.9 | 121.2 | 116.5 | Means | 140.4 | 130.4 |
|  | Sdevs | 19.34 | 21.06 | 24.33 | 21.59 | Sdevs | 22.33 | 14.60 |
| **Na** (mmol/L) | (n) | 10 | 10 | 10 | 10 | (n) | 10 | 10 |
|  | Means | 144.9 | 145.6 | 145.7 | 144.9 | Means | 142.8 | 142.6 |
|  | Sdevs | 0.57 | 1.26 | 1.06 | 0.88 | Sdevs | 1.10 | 1.14 |
| **Cl** (mmol/L) | (n) | 10 | 10 | 10 | 10 | (n) | 10 | 10 |
|  | Means | 101.9 | 102.2 | 102.5 | 103.0 | Means | 103.2 | 102.8 |
|  | Sdevs | 1.10 | 1.48 | 1.18 | 1.05 | Sdevs | 2.17 | 0.84 |

*, P < 0.05

**Supplementary Table 6. Organ weight analysis in general toxicity study (Male)**

| **Rat/Athymic nude rat** | | **Male** | | | | | | |
| --- | --- | --- | --- | --- | --- | --- | --- | --- |
|  |  | **Acute** | | | |  | **Chronic** | |
|  |  | **Vehicle** | **Low** | **Medium** | **High** |  | **Vehicle** | **High** |
|  |  | 0 | 1 × 10^5^ | 1 × 10^6^ | 3 × 10^6^ |  | 0 | 3 × 10^6^ |
| **TBW** (g) | (n) | 10 | 10 | 10 | 10 | (n) | 5 | 5 |
|  | Means | 274.7 | 274.3 | 273.8 | 276.1 | Means | 403.2 | **352.2*** |
|  | Sdevs | 20.94 | 17.62 | 16.83 | 17.92 | Sdevs | 33.38 | 24.72 |
| **Adrenal glands** (g) | (n) | 10 | 10 | 10 | 10 | (n) | 5 | 5 |
|  | Means | 0.059 | 0.063 | 0.062 | 0.061 | Means | 0.067 | 0.061 |
|  | Sdevs | 0.0081 | 0.0083 | 0.0052 | 0.0065 | Sdevs | 0.0058 | 0.0040 |
| **Brain** (g) | (n) | 10 | 10 | 10 | 10 | (n) | 5 | 5 |
|  | Means | 1.798 | 1.767 | 1.759 | 1.780 | Means | 1.893 | 1.809 |
|  | Sdevs | 0.0676 | 0.0505 | 0.0287 | 0.0716 | Sdevs | 0.0854 | 0.0675 |
| **Epididymis** (g) | (n) | 10 | 10 | 10 | 10 | (n) | 5 | 5 |
|  | Means | 0.807 | 0.797 | 0.785 | 0.791 | Means | 1.090 | 1.003 |
|  | Sdevs | 0.1429 | 0.1035 | 0.0554 | 0.0568 | Sdevs | 0.0950 | 0.1741 |
| **Heart** (g) | (n) | 10 | 10 | 10 | 10 | (n) | 5 | 5 |
|  | Means | 0.957 | 1.024 | 1.012 | 0.996 | Means | 1.183 | 1.106 |
|  | Sdevs | 0.0812 | 0.0935 | 0.0589 | 0.0531 | Sdevs | 0.1699 | 0.1311 |
| **Kidneys** (g) | (n) | 10 | 10 | 10 | 10 | (n) | 5 | 5 |
|  | Means | 2.328 | 2.312 | 2.380 | 2.383 | Means | 2.889 | **2.457*** |
|  | Sdevs | 0.2725 | 0.1472 | 0.1525 | 0.1065 | Sdevs | 0.2948 | 0.2014 |
| **Liver** (g) | (n) | 10 | 10 | 10 | 10 | (n) | 5 | 5 |
|  | Means | 9.978 | 9.738 | 9.850 | 10.063 | Means | 12.110 | **10.731*** |
|  | Sdevs | 1.3714 | 0.9610 | 0.8357 | 0.7875 | Sdevs | 0.7955 | 0.8348 |
| **Lung with bronchi** (g) | (n) | 10 | 10 | 10 | 10 | (n) | 5 | 5 |
|  | Means | 1.218 | 1.250 | 1.336 | 1.330 | Means | 1.536 | 1.468 |
|  | Sdevs | 0.1719 | 0.1162 | 0.2907 | 0.1176 | Sdevs | 0.2156 | 0.2649 |
| **Pituitary gland** (g) | (n) | 10 | 10 | 10 | 10 | (n) | 5 | 5 |
|  | Means | 0.008 | 0.008 | 0.008 | 0.009 | Means | 0.010 | 0.008 |
|  | Sdevs | 0.0013 | 0.0007 | 0.0013 | 0.0014 | Sdevs | 0.0019 | 0.0011 |
| **Prostate** (g) | (n) | 10 | 10 | 10 | 10 | (n) | 5 | 5 |
|  | Means | 0.275 | 0.232 | 0.280 | 0.267 | Means | 0.394 | 0.372 |
|  | Sdevs | 0.0767 | 0.0884 | 0.0605 | 0.0670 | Sdevs | 0.1312 | 0.0881 |
| **Salivary galnds** (g) | (n) | 10 | 10 | 10 | 10 | (n) | 5 | 5 |
|  | Means | 0.651 | 0.645 | 0.672 | 0.655 | Means | 0.794 | 0.706 |
|  | Sdevs | 0.0778 | 0.0519 | 0.0553 | 0.0498 | Sdevs | 0.1540 | 0.0464 |
| **Seminal vesicles with coagulating gland** (g) | (n) | 10 | 10 | 10 | 10 | (n) | 5 | 5 |
|  | Means | 1.071 | 1.042 | 1.075 | 1.048 | Means | 1.878 | 1.532 |
|  | Sdevs | 0.2162 | 0.2824 | 0.1863 | 0.2132 | Sdevs | 0.3238 | 0.2487 |
| **Spleen** (g) | (n) | 10 | 10 | 10 | 10 | (n) | 5 | 5 |
|  | Means | 0.552 | 0.532 | 0.567 | 0.628 | Means | 0.683 | 0.618 |
|  | Sdevs | 0.0513 | 0.0536 | 0.0424 | 0.2297 | Sdevs | 0.0963 | 0.0304 |
| **Testes** (g) | (n) | 10 | 10 | 10 | 10 | (n) | 5 | 5 |
|  | Means | 2.664 | 2.705 | 2.752 | 2.640 | Means | 2.895 | 2.511 |
|  | Sdevs | 0.2509 | 0.0965 | 0.1802 | 0.1521 | Sdevs | 0.1555 | 0.7471 |
| **Thyroid and parathyroid glands** (g) | (n) | 10 | 10 | 10 | 10 | (n) | 5 | 5 |
|  | Means | 0.017 | 0.018 | 0.020 | 0.019 | Means | 0.023 | **0.018*** |
|  | Sdevs | 0.0036 | 0.0032 | 0.0039 | 0.0030 | Sdevs | 0.0021 | 0.0033 |

*, P < 0.05

**Supplementary Table 6. Organ weight analysis in general toxicity study (Female)**

| **Rat/Athymic nude rat** | | **Female** | | | | | | |
| --- | --- | --- | --- | --- | --- | --- | --- | --- |
|  |  | **Acute** | | | |  | **Chronic** | |
|  |  | **Vehicle** | **Low** | **Medium** | **High** |  | **Vehicle** | **High** |
|  |  | 0 | 1 × 10^5^ | 1 × 10^6^ | 3 × 10^6^ |  | 0 | 3 × 10^6^ |
| **TBW** (g) | (n) | 10 | 10 | 10 | 10 | (n) | 5 | 5 |
|  | Means | 175.5 | 182.0 | 180.6 | 178.1 | Means | 208.8 | 224.4 |
|  | Sdevs | 9.27 | 20.88 | 16.38 | 9.53 | Sdevs | 34.43 | 10.06 |
| **Adrenal glands** (g) | (n) | 10 | 10 | 10 | 10 | (n) | 5 | 5 |
|  | Means | 0.083 | 0.084 | 0.084 | 0.082 | Means | 0.090 | 0.083 |
|  | Sdevs | 0.0125 | 0.0110 | 0.0058 | 0.0083 | Sdevs | 0.0206 | 0.0061 |
| **Brain** (g) | (n) | 10 | 10 | 10 | 10 | (n) | 5 | 5 |
|  | Means | 1.644 | 1.668 | 1.647 | 1.648 | Means | 1.727 | 1.721 |
|  | Sdevs | 0.0492 | 0.0583 | 0.0567 | 0.0328 | Sdevs | 0.0508 | 0.0462 |
| **Epididymis** (g) | (n) | 10 | 10 | 10 | 10 | (n) | 5 | 5 |
|  | Means | 0.680 | 0.684 | 0.706 | 0.731 | Means | 0.855 | 0.774 |
|  | Sdevs | 0.0580 | 0.0540 | 0.0613 | 0.1039 | Sdevs | 0.0730 | 0.0517 |
| **Heart** (g) | (n) | 10 | 10 | 10 | 10 | (n) | 5 | 5 |
|  | Means | 1.496 | 1.559 | 1.524 | 1.509 | Means | 1.698 | 1.723 |
|  | Sdevs | 0.1135 | 0.1279 | 0.1472 | 0.0944 | Sdevs | 0.1511 | 0.0716 |
| **Kidneys** (g) | (n) | 10 | 10 | 10 | 10 | (n) | 5 | 5 |
|  | Means | 6.228 | 6.602 | 6.362 | 6.170 | Means | 8.583 | 7.409 |
|  | Sdevs | 0.6125 | 0.7845 | 0.7036 | 0.5620 | Sdevs | 1.9407 | 0.6932 |
| **Liver** (g) | (n) | 10 | 10 | 10 | 10 | (n) | 5 | 5 |
|  | Means | 1.009 | 0.977 | 1.028 | 1.059 | Means | 1.056 | 1.079 |
|  | Sdevs | 0.0932 | 0.0660 | 0.1083 | 0.2075 | Sdevs | 0.0706 | 0.0866 |
| **Lung with bronchi** (g) | (n) | 10 | 10 | 10 | 10 | (n) | 5 | 5 |
|  | Means | 0.085 | 0.075 | 0.880 | 0.074 | Means | 0.084 | 0.088 |
|  | Sdevs | 0.0189 | 0.0101 | 0.0075 | 0.0102 | Sdevs | 0.0175 | 0.0075 |
| **Pituitary gland** (g) | (n) | 10 | 10 | 10 | 10 | (n) | 5 | 5 |
|  | Means | 0.009 | 0.008 | 0.008 | **0.008*** | Means | 0.011 | 0.011 |
|  | Sdevs | 0.0007 | 0.0013 | 0.0015 | 0.0020 | Sdevs | 0.0014 | 0.0021 |
| **Prostate** (g) | (n) | 10 | 10 | 10 | 10 | (n) | 5 | 5 |
|  | Means | 0.454 | 0.437 | 0.439 | 0.479 | Means | 0.505 | 0.518 |
|  | Sdevs | 0.0567 | 0.0592 | 0.0488 | 0.1494 | Sdevs | 0.1323 | 0.0542 |
| **Salivary galnds** (g) | (n) | 10 | 10 | 10 | 10 | (n) | 5 | 5 |
|  | Means | 0.418 | 0.422 | 0.423 | 0.416 | Means | 0.912 | 0.491 |
|  | Sdevs | 0.0486 | 0.0527 | 0.0588 | 0.0569 | Sdevs | 0.9185 | 0.0277 |
| **Seminal vesicles with coagulating gland** (g) | (n) | 10 | 10 | 10 | 10 | (n) | 5 | 5 |
|  | Means | 0.014 | 0.013 | 0.014 | 0.012 | Means | 0.014 | 0.014 |
|  | Sdevs | 0.0026 | 0.0017 | 0.0020 | 0.0017 | Sdevs | 0.0035 | 0.0019 |
| **Spleen** (g) | (n) | 10 | 10 | 10 | 10 | (n) | 5 | 5 |
|  | Means | 0.585 | 0.538 | 0.407 | 0.451 | Means | 0.542 | 0.572 |
|  | Sdevs | 0.3152 | 0.2118 | 0.1467 | 0.2566 | Sdevs | 0.2986 | 0.2973 |
| **Testes** (g) | (n) | 10 | 10 | 10 | 10 | (n) | 5 | 5 |
|  | Means | 175.5 | 182.0 | 180.6 | 178.1 | Means | 208.8 | 224.4 |
|  | Sdevs | 9.27 | 20.88 | 16.38 | 9.53 | Sdevs | 34.43 | 10.06 |
| **Thyroid and parathyroid glands** (g) | (n) | 10 | 10 | 10 | 10 | (n) | 5 | 5 |
|  | Means | 0.083 | 0.084 | 0.084 | 0.082 | Means | 0.090 | 0.083 |
|  | Sdevs | 0.0125 | 0.0110 | 0.0058 | 0.0083 | Sdevs | 0.0206 | 0.0061 |

*, P < 0.05

**Supplementary materials and methods**

**Flow Cytometric Analysis**

AhNSCs were harvested using 0.25% trypsin-EDTA (Gibco, Grand Island, NY, USA) and transferred into fluorescence-activated cell sorting (FACS) tubes (BD Bioscience, Franklin Lakes, NJ, USA). For cell surface marker staining, monoclonal antibodies against CD29 (1:100, BD Bioscience), CD44 (1:100, BD Bioscience), CD140b (1:100, BD Bioscience), CD11b (1:100, BD Bioscience), HLA-DR (1:100, BD Bioscience), CD34 (1:100, BD Bioscience), CD45 (1:100, BD Bioscience), CD19 (1:100, BD Bioscience)), or CD31(1:100, BD Bioscience) were added to the tubes and incubated in the dark at room temperature (RT). After 20 minutes (mins), FACS buffer (BD Biosciences) was added to thetubes, followed by centrifugation at 500 g for 3 mins. The cells were then resuspended in Dulbecco’s phosphate-buffered saline (DPBS) containing 4% paraformaldehyde (PFA, Biosesang, Gyeonggi, South Korea). For intracellular staining, AhNSCs fixed with DPBS containing 4% PFA were permeabilized with 0.1% Triton X-100 for 10 mins and incubated with a monoclonal antibody against Nestin (1:100, BD Bioscience) in the dark at RT for 30 mins. Fluorescence intensity was measured using a FACSCalibur (Becton Dickinson, San Jose, CA, USA), and data analysis was performed using FLOWJO software (Tree Star, Inc., Ashland, OR, USA).

**Immunocytochemistry**

For differentiation, AhNSCs were cultured in DMEM/F12 (Gibco) supplemented with 1% B27 supplement (Xenofree, Gibco), 1% N-2 supplement (Gibco), 5 µg/mL gentamicin (Gibco), 0.5% FBS (Gibco), and 0.5 mM 3-isobutyl-1-methylxanthine (IBMX) (Sigma, St. Louis, MO, USA) for 3 days. After differentiation, AhNSCs were fixed in ice-cold DPBS containing 4% PFA at RT for 20 mins, followed by permeabilization with 0.2% Triton X-100 for 20 mins. After permeabilization, the cells were blocked with PBS containing 2% Normal goat serum (NGS, Abcam, Cambridge, MA, USA) and 1% Bovine serum albumin (BSA, GenDEPOT, Barker, TX, USA). The cells were then incubated overnight at 4°C with the following primary antibodies: Nestin (1:200, Novus Biologicals, Littleton, CO, USA), Tuj1 (1:1000, Abcam), GFAP (1:2000, Abcam), or Oligodendrocyte 1 (1:250, Millipore, Temecula, CA, USA). After washing, the samples were treated with Alexa Fluor 594-conjugated goat anti-Rabbit IgG secondary antibody (1:500, Abcam) and counterstained with DAPI for 10 mins at RT. After mounting, the cells were visualized using a confocal laser scanning microscopy (BIORP, Leica, Wetzlar, Germany).

**Immunohistochemistry (IHC)**

Deparaffinized and rehydrated sections were boiled with target retrieval solution (Dako, Carpentaria, CA, USA) in a microwave for 5 mins four times. The slides were then incubated in 0.3% ammonia in 70% methanol for 1 hour (hr). Next, the slides were washed in 50% methanol for 10 mins before being treated with 3% hydrogen peroxide in methanol for 12 mins to quench endogenous peroxidase activities. Protein blocking was performed by incubating the slides in PBS containing 5%NGS (Abcam) and 2% BSA (GenDEPOT) for 1 hr at RT. Primary antibodies were treated overnight at 4°C; GFAP (1:2000, Abcam); NeuN (1:500, Millipore); Tuj1 (1:1000, Abcam); CD31(1:2000, Abcam); Iba1 (1:1000, Abcam). The slides were then incubated with appropriate HRP-conjugated secondary antibody (Abcam) at RT for 1 hr and then reacted with DAB (3,3’-diaminobenzidine tetrahydrochloride). Nuclei were counterstained with hematoxylin. Stained sections were scanned. The colors in the scanned images were separated into purple and brown using QuPath (Queen’s University Belfast, UK). Computational color deconvolution was applied to separate the hematoxylin(purple) and DAB (brown) staining. The brown immunohistochemical staining was analyzed using ImageJ software (NIH Image, Bethesda, MD, USA).

**Preclinical general toxicity test**

The food consumption per cage was measured. The food amount was weighed and applied for each cage. After 4-7 days, the food amount was weighed. The average daily intake per animal (g/animal/day) was calculated. In hematology analysis, approximately 1.5 mL of blood was collected from animals scheduled for planned euthanasia. About 0.5 mL of the blood was placed in a collection tube containing the anticoagulant EDTA-2K for hematological analysis. The remaining 1.0 mL of blood was placed in a collection tube containing 3.2% sodium citrate, then centrifuged (at around 3000 rpm for 10 mins at RT) to separate the plasma, which was used for coagulation time testing.Total leukocyte count (WBC), mean corpuscular hemoglobin (MCH), total red blood cell count (RBC), mean corpuscular hemoglobin concentration (MCHC), Hemoglobin (HGB), platelet count (PLT), Hematocrit (HCT), Reticulocyte count, mean corpuscular volume (MCV), and WBC differential count were analyzed by an ADVIA2120i hematology analyzer (Siemens, Washington, D.C., USA). Prothrombin time (PT) and activated partial thromboplastin time (APTT) were measured by an ACL Elite Pro coagulation analyzer (Instrumentation Laboratory, Milano, Italy). In clinical chemistry analysis, approximately 1.5 mL of blood was collected from animals scheduled for planned euthanasia and placed in a tube without anticoagulant. The blood was then left at RT for at least 90 mins before being centrifuged (at around 3000 rpm for 10 mins at RT) to separate the serum. The serum was performed using a Toshiba 200FR NEO chemistry analyzer (Toshiba Co., Tokyo, Japan).
